# Genetic evidence strengthens the bidirectional connection between gut microbiota and *Shigella* infection: insights from a two-sample Mendelian randomization study

**DOI:** 10.3389/fmicb.2024.1361927

**Published:** 2024-03-01

**Authors:** Jingyi Peng, Kun Cai, Guanglei Chen, Linxiao Liu, Lili Peng

**Affiliations:** ^1^Guizhou University of Traditional Chinese Medicine, Guiyang, Guizhou, China; ^2^The First People’s Hospital of Hangzhou Lin’an District, Hangzhou, Zhejiang, China

**Keywords:** gut microbiota, *Shigella* infections, Mendelian randomization, pathogenic bacterial colonization, antagonistic effects

## Abstract

**Background:**

In recent investigations, substantial strides have been made in the precise modulation of the gut microbiota to prevent and treat a myriad of diseases. Simultaneously, the pressing issue of widespread antibiotic resistance and multidrug resistance resulting from *Shigella* infections demands urgent attention. Several studies suggest that the antagonistic influence of the gut microbiota could serve as a novel avenue for impeding the colonization of pathogenic microorganisms or treating *Shigella* infections. However, conventional research methodologies encounter inherent challenges in identifying antagonistic microbial agents against *Shigella*, necessitating a comprehensive and in-depth analysis of the causal relationship between *Shigella* infections and the gut microbiota.

**Materials and methods:**

Utilizing the aggregated summary statistics from Genome-Wide Association Studies (GWAS), we conducted Mendelian Randomization (MR) analyses encompassing 18,340 participants to explore the interplay between the gut microbiota and *Shigella* infections. This investigation also involved 83 cases of *Shigella* infection patients and 336,396 control subjects. In the positive strand of our findings, we initially performed a preliminary analysis using the Inverse Variance Weighting (IVW) method. Subsequently, we undertook sensitivity analyses to assess the robustness of the results, addressing confounding factors’ influence. This involved employing the Leave-One-Out method and scrutinizing funnel plots to ensure the reliability of the MR analysis outcomes. Conclusively, a reverse MR analysis was carried out, employing the Wald ratio method due to the exposure of individual Single Nucleotide Polymorphisms (SNPs). This was undertaken to explore the plausible associations between *Shigella* infections and genetically predicted compositions of the gut microbiota.

**Results:**

In this study, we employed 2,818 SNPs associated with 211 species of gut microbiota as instrumental variables (IVs). Through IVW analysis, our positive MR findings revealed a significant negative correlation between the occurrence of *Shigella* infections and the phylum Tenericutes (OR: 0.18, 95% CI: 0.04–0.74, *p* = 0.02), class Mollicutes (OR: 0.18, 95% CI: 0.04–0.74, p = 0.02), genus *Intestinimonas* (OR: 0.16, 95% CI: 0.04–0.63, *p* = 0.01), genus *Gordonibacter* (OR: 0.39, 95% CI: 0.16–0.93, *p* = 0.03), and genus *Butyrivibrio* (OR: 0.44, 95% CI: 0.23–0.87, *p* = 0.02). Conversely, a positive correlation was observed between the occurrence of *Shigella* infections and genus *Sutterella* (OR: 10.16, 95% CI: 1.87–55.13, *p* = 0.01) and genus *Alistipes* (OR: 12.24, 95% CI: 1.71–87.34, *p* = 0.01). In sensitivity analyses, utilizing MR-Egger regression analysis and MR Pleiotropy Residual Sum and Outlier (MR-PRESSO) detection, all outcomes demonstrated robust stability. Simultaneously, in the reverse MR analysis, *Shigella* infections resulted in an upregulation of four bacterial genera and a downregulation of three bacterial genera.

**Conclusion:**

In summation, the MR analysis outcomes corroborate the presence of bidirectional causal relationships between the gut microbiota and *Shigella* infections. This study not only unveils novel perspectives for the prevention and treatment of *Shigella* infections but also furnishes fresh insights into the mechanistic underpinnings of how the gut microbiota contributes to the pathogenesis of *Shigella* infections. Consequently, the established dual causal association holds promise for advancing our understanding and addressing the complexities inherent in the interplay between the gut microbiota and *Shigella* infections, thereby paving the way for innovative therapeutic interventions and preventive strategies in the realm of *Shigella*-related diseases.

## Introduction

*Shigella* infection, a clinical syndrome induced by the invasion of *Shigella* bacteria into the terminal ileum, colon, and rectal epithelium, manifests prominently with symptoms such as recurrent bloody stools, fever, diarrhea, weight loss, and abdominal spasms (Kotloff et al., 2018). *Shigella*, classified as Gram-negative short rods, currently comprises four species: *Shigella dysenteriae*, *Shigella sonnei*, *Shigella boydii*, and *Shigella flexneri* (Muzembo et al., 2023). This highly transmissible pathogen spreads through various means, including food, water, insect vectors, or direct fecal-oral transmission (Barry and Levine, 2019; Muzembo et al., 2023). Globally, low-income and middle-income countries witness an annual toll of over 200,000 deaths attributed to *Shigella* infection, with children under the age of five accounting for a staggering 30% (Khalil et al., 2018). In the United Kingdom, there has been an outbreak of multidrug-resistant *Shigella sonnei* among men who engage in sexual activities with other men (MSM). This outbreak has raised concerns due to the presence of extensively drug-resistant strains, highlighting the challenges posed by antimicrobial resistance in the field of public health. To better understand the genetic epidemiology of this outbreak, researchers conducted genomic analyses on isolates collected from various countries including the UK, Australia, Belgium, France, and the USA (Tansarli et al., 2023). The results of these analyses revealed a strain of *Shigella sonnei* that is not only multidrug-resistant but also shares a common resistance plasmid, indicating a potential international spread of this resistant strain (Mason et al., 2023; Thorley et al., 2023). Consequently, this underscores the urgent need for enhanced global surveillance and collaborative efforts in sharing genomic data to effectively combat the spread of antimicrobial resistance. In addition to the high incidence and mortality associated with *Shigella* infection, recurrent episodes contribute to prolonged distress for affected individuals, leading to complications such as delayed childhood development and cognitive impairments (Kotloff and Chisti, 2020). Given the incomplete understanding of therapeutic approaches for *Shigella* infection, exploring alternative effective treatments and identifying drug targets assumes paramount significance in the prevention and control of *Shigella*-related illnesses. Consequently, comprehensive research on treatment modalities for *Shigella* infection is imperative.

In recent years, the profound investigation into the gut microbiota has ushered in a maturation of strategies aimed at precision modulation for the prevention and treatment of various diseases (Cai et al., 2022). Throughout the pathogenic progression of *Shigella* infections, encompassing diverse pathogens, including *Shigella dysenteriae*, these organisms adeptly exploit various intestinal signals to augment their pathogenicity (Chowdhury et al., 2023). The competition among gut microbiota is crucial for the colonization of intestinal bacterial pathogens in the gut environment. Co-infection of *Entamoeba histolytica* and enteric pathogens leads to immune activation, epithelial cell damage, and increased virulence, possibly due to enhanced toxicity following bacterial engulfment by *Entamoeba histolytica*. Such co-infections have been reported in regions endemic for these pathogens, particularly among children with diarrheal diseases co-infected with *Entamoeba histolytica* and enteropathogenic *Escherichia coli* (EPEC) or *Shigella dysenteriae* (Bhagchandani et al., 2023; Jaswal et al., 2023). Concurrently, an array of studies underscores the antagonistic potential of the gut microbiota, a phenomenon capable of countering the colonization of exogenous pathogens, thereby presenting a novel avenue for *Shigella* infection management (Xerri and Payne, 2022). In certain animal experiments, the therapeutic efficacy and safety of mixed probiotics in treating *Shigella dysenteriae* infection in albino rats have shown promising results. Our study contributes to the enrichment of this therapeutic strategy (Ibejekwe et al., 2023). Nevertheless, the precise correlation between the onset of *Shigella* infections and the gut microbiota remains incompletely elucidated. The quest for microbial entities exerting antagonistic effects against *Shigella* encounters inherent complexities, thereby necessitating an urgent and comprehensive causal relationship analysis between *Shigella* infections and the gut microbiota.

Mendelian Randomization (MR), rooted in Mendel’s laws of inheritance and the principles of Instrumental Variables (IVs), stands as a robust statistical methodology. By employing genetic data as mediators, MR effectively unravels causal relationships between exposure risk factors and outcomes, thereby adeptly mitigating the interference of confounding factors (Davey Smith and Ebrahim, 2003). In this study, the application of MR analysis seeks to delve into the bidirectional causal relationships between the human gut microbiota and *Shigella* infections. When selecting Single Nucleotide Polymorphisms (SNPs) as IVs, certain criteria must be met: (a) the assumption of relevance, ensuring a strong correlation between each IV and the exposure factor; (b) the assumption of independence, asserting that each IV remains uncorrelated with other potential confounding factors; (c) the assumption of exclusivity, stipulating that each IV singularly influences the outcome through the exposure factor. These stringent conditions underpin the validity and integrity of the MR analysis, assuring a meticulous exploration of the intricate interplay between genetic determinants and the complex dynamics of the gut microbiota in relation to *Shigella* infections.

## Materials and methods

### Study design

**Figure 1:**
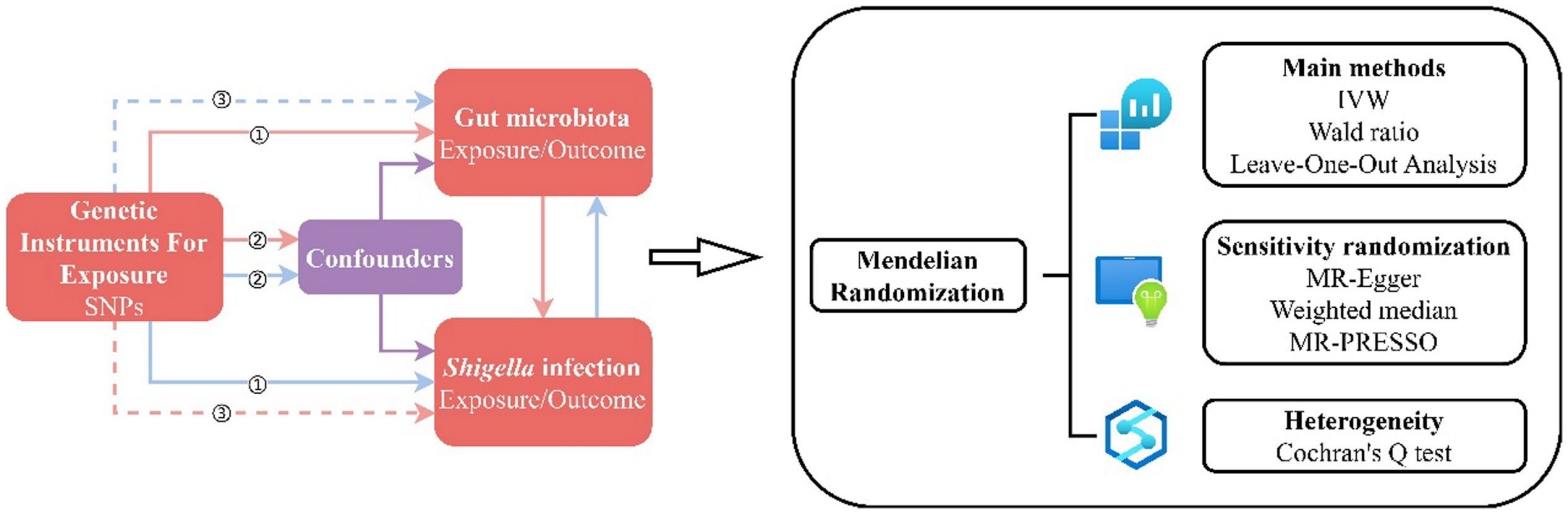
MR Design Flowchart. The schematic representation delineates the workflow of our MR study, adhering to three critical assumptions: ① Each Instrumental Variable (IV) exhibits a robust correlation with the exposure factor; ② Each IV remains impervious to the influence of confounding factors; ③ Each IV singularly influences the outcome exclusively through the exposure factor. The red solid lines depict the forward MR analysis, examining the potential causal links between the human gut microbiota as the exposure and *Shigella* infections as the outcome. Conversely, the blue solid lines signify the reverse MR analysis, exploring the reciprocal causality between *Shigella* infections as the exposure and the human gut microbiota as the outcome.

The present investigation employs MR analysis, seeking to elucidate putative causal relationships between the gut microbiota and *Shigella* infections. We utilized summary statistics from Genome-Wide Association Studies (GWAS) sourced from two public databases, MiBioGen and FinnGen. Employing a two-sample MR analysis, we assessed 211 taxonomic units encompassing 9 phyla, 16 classes, 20 orders, 35 families, and 131 genera, representing a comprehensive spectrum of bacterial classifications. This served as the exposure factor in exploring potential causal links with *Shigella* infections as the designated outcome (Davey Smith and Hemani, 2014). Moreover, a reverse causal relationship study was conducted to complement our primary analyses. The flowchart in Figure 1 illustrates the procedural framework of this MR investigation. Throughout the entirety of the analytical process, in order to ensure the robustness and transparency of the MR methodology, we rigorously adhered to the guidelines outlined in the MR Strengthening the Reporting of Observational Studies in Epidemiology (MRSTROBE) statement (Skrivankova et al., 2021).

### Data sources

The dataset underpinning our investigation emanates from the MiBioGen database, a preeminent repository in the landscape of host-genetics-microbiome correlation studies. This resource represents an epitome of comprehensiveness, encompassing 24 population cohorts spanning 11 countries and diverse ethnicities (MiBioGen Consortium Initiative et al., 2018; Kurilshikov et al., 2021), aggregating a total of 18,340 participants exclusively of European descent (Li et al., 2023; Supplementary material 1-S1).

The data pertaining to *Shigella* infection were sourced from the FinnGen database. Our investigation drew from the most recent FinnGen study, encompassing 83 afflicted individuals and 336,396 control subjects, in order to extract IVs genetically linked to *Shigella* infection (Liu et al., 2022). This utilization of FinnGen as a foundational dataset affords a comprehensive genetic perspective, given its extensive coverage of the Finnish population. The cohort’s significance is underscored by the substantial number of individuals involved, contributing to a robust and statistically meaningful analysis. FinnGen represents a pivotal biobank initiative involving the genotyping of half a million Finnish individuals (Kurki et al., 2023). The definition of *Shigella* infection aligns with the International Classification of Diseases (ICD) coding system, specifically ICD-10 (A03) and ICD-9 (004; Supplementary material 1-S2).

### Instrumental variable selection

Our investigation undertook a comprehensive analysis of 211 bacterial taxonomic units across five hierarchical levels (phylum, class, order, family, genus).

In investigating the intestinal microbiota as an exposure factor and *Shigella* infection as the outcome, meticulous criteria were applied to the IVs to ensure the stability of research data and the precision of results. These criteria are outlined as follows: (a) The significance threshold for IVs associated with the intestinal microbiota at the genome-wide level should meet *p* < 1 × 10^−5^ (Cheng et al., 2020). (b) To fulfill the prerequisites for MR analysis, linkage disequilibrium (LD) analysis was conducted based on the European 1,000 Genomes Project. It was mandated that the R^2^ of IVs be less than 0.001, with LD limited to 10,000 kb. (c) In order to mitigate the potential influence of allelic variants on the causal relationship between the intestinal microbiota and *Shigella* infection, we utilized the F-statistic to assess the strength of genetic variation as IVs. Variants with an F-statistic of ≤10 were deemed weak IVs, indicative of potential analytical bias. Conversely, an F-statistic exceeding 10 signified a robust instrumental variable; consequently, IVs with an F-statistic below 10 were excluded (Burgess et al., 2017b). By rigorously enforcing these criteria, we aimed to fortify the causal inferences drawn from our MR framework, thereby enhancing the reliability and robustness of our study findings.

In the pursuit of reverse MR analysis, stringent criteria were imposed upon the IVs associated with *Shigella* infection. These criteria necessitated that the *p*-value be less than 1 × 10^−5^, the R^2^ of IVs be below 0.001, and the LD be restricted to 10,000 kb. Similarly, IVs with an F-statistic below 10 were systematically excluded from the analysis.

### MR analysis and sensitivity analysis

The essential dataset for our investigation was acquired from publicly accessible repositories, namely FinnGen and MiBioGen. Subsequently, a comprehensive bidirectional MR analysis was undertaken to interrogate the intricate relationship between exposure variables and their corresponding outcomes. Within the MR analytical framework, the primary computational tool employed was R (version 4.3.1), further enhanced by the utilization of the “Two-Sample MR” R package (version 0.5.7; Mounier and Kutalik, 2023).

The coefficient R^2^ is employed as a metric to signify the proportion of phenotypic variance elucidated by SNPs. The computational formula for R^2^ is delineated as follows:

$$R^{2}=2\times {\mathrm{Beta}}^{2}\times EAF\times \frac{\left(1-EAF\right)}{\begin{array}{c} 2\times {\mathrm{Beta}}^{2}\times EAF\times \left(1-EAF\right)\\ +{SE}^{2}\times 2\times \mathrm{Sample}\;\mathrm{size}\times EAF\times \left(1-EAF\right) \end{array}}$$

Where β signifies the effect size, EAF represents the effect allele frequency, and SE denotes the standard error of the effect size. This metric offers valuable insights into the proportion of phenotypic variability attributed to the considered SNPs. To gauge the robustness of the IVs, the F-statistic is calculated, defined by the equation:

$$F=R^{2}\times \frac{\left(\mathrm{Sample}\;\mathrm{size}-1-k\right)}{\left(1-R^{2}\right)\times k}$$

Here, k denotes the number of SNPs encompassed in the instrument. Notably, a threshold F-statistic exceeding 10 is conventionally deemed to hold significant statistical implications (Zuber et al., 2020), signifying a robust instrument and ensuring that causal inferences remain unaffected by bias-induced deviations (Palmer et al., 2012).

To validate the effectiveness of all IVs, the Inverse Variance Weighted (IVW) method was initially employed, generating a weighted overall effect based on the significance of *p*-values (Brion et al., 2013). Heterogeneity among IVs was assessed using Cochran’s Q test, with significance attributed to a *p*-value below 0.05 (Aslam, 2023).

In order to ensure the stability of IVW results and circumvent biases arising from ineffective IVs, a sensitivity analysis was conducted. This encompassed MR-Egger, Weighted Median (WM), and MR Pleiotropy Residual Sum and Outlier (MR-PRESSO) methods (Burgess et al., 2017a). MR-Egger, incorporating directional pleiotropy tests, causal effect tests, and causal effect estimation, assessed the potential pleiotropy of genetic variations, providing consistent estimates of causal effects under the weaker InSIDE (Independent of Direct Effect) assumption (Burgess and Thompson, 2017). WM facilitated precise estimation of causal relationships. MR-PRESSO, through outlier detection and removal of anomalous IVs, enhanced the robustness of the analysis (Fan et al., 2023). Results were deemed more reliable when effect sizes between IVW and sensitivity analysis were consistent, and the *p*-value was below 0.05. Lastly, a reverse MR analysis was undertaken to evaluate the potential existence of a reciprocal causal relationship between the gut microbiota and *Shigella* infection.

In addition, we employed a leave-one-out analysis to fortify the robustness of our MR outcomes, as the exclusion of any individual Instrumental Variable (IV) failed to significantly alter the results. This additional analytical step, utilizing a leave-one-out approach, serves as a compelling validation of the stability and reliability of our MR findings.

## Results

Through a rigorous series of quality control measures, we ultimately identified 2,818 SNPs associated with 211 bacterial species to serve as IVs. The F-statistics for the gut microbiota ranged from 14.58 to 88.42, with an average of 21.69, consistently surpassing the threshold of 10. This observation indicates a diminished likelihood of bias occurrence, affirming the robustness of our instrumental variable selection.

### Causal effects of gut microbiota on *Shigella* infection

**Figure 2:**
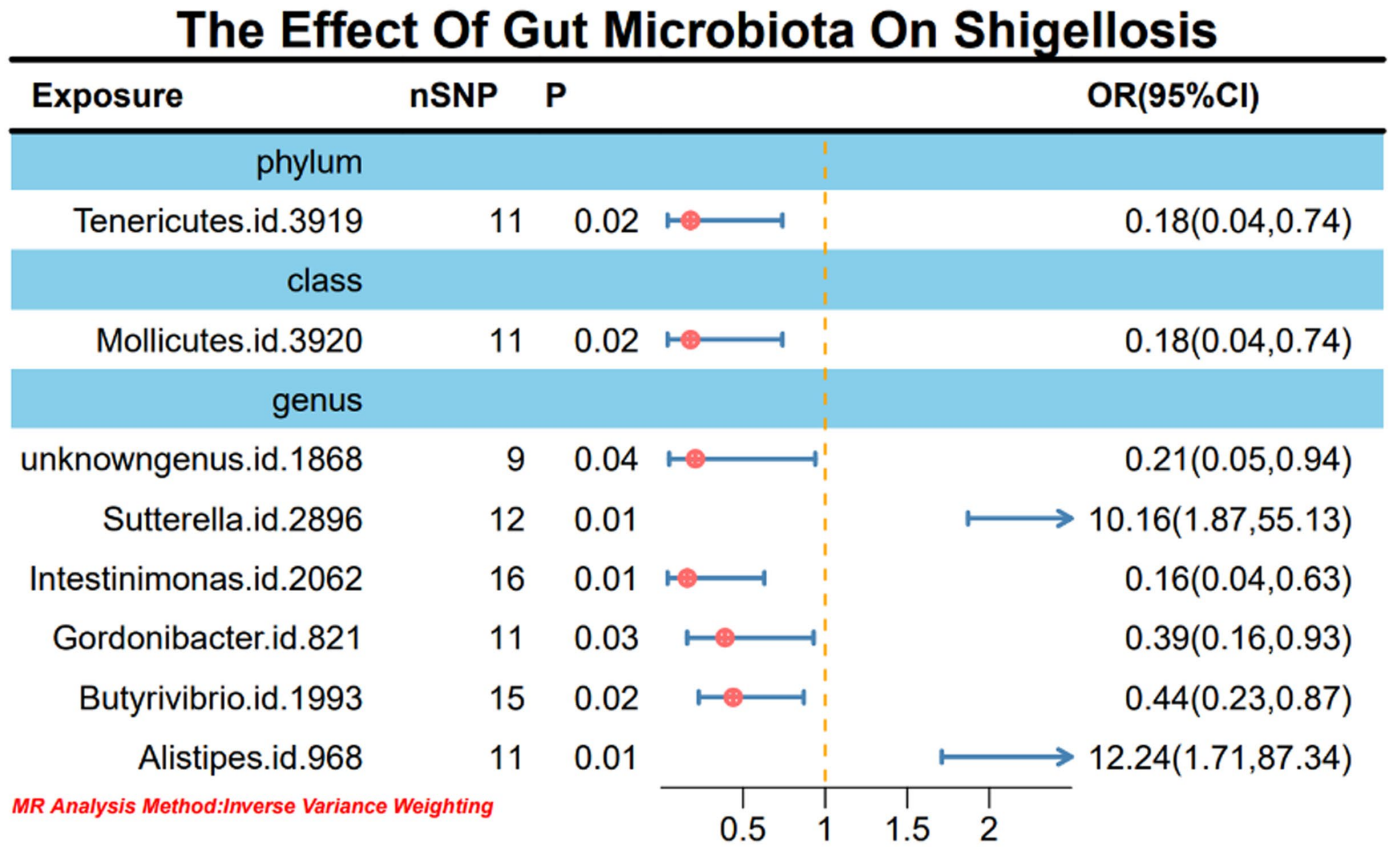
MR forest plot analysis illustrates the causal effects of gut microbiota on *Shigella* infection.

Upon the absence of conspicuous heterogeneity following Cochran’s Q test, our initial IVW analysis revealed significant associations. Specifically, the phylum Tenericutes (OR: 0.18, 95% CI: 0.04–0.74, *p* = 0.02), class Mollicutes (OR: 0.18, 95% CI: 0.04–0.74, *p* = 0.02), genus *Sutterella* (OR: 10.16, 95% CI: 1.87–55.13, *p* = 0.01), genus *Intestinimonas* (OR: 0.16, 95% CI: 0.04–0.63, *p* = 0.01), genus *Gordonibacter* (OR: 0.39, 95% CI: 0.16–0.93, *p* = 0.03), genus *Butyrivibrio* (OR: 0.44, 95% CI: 0.23–0.87, *p* = 0.02), and genus *Alistipes* (OR: 12.24, 95% CI: 1.71–87.34, *p* = 0.01) exhibited significant causal relationships with *Shigella* infection (Figure 2; Supplementary material 1-S3).

### Causal effects of *Shigella* infection on the gut microbiota

**Figure 3:**
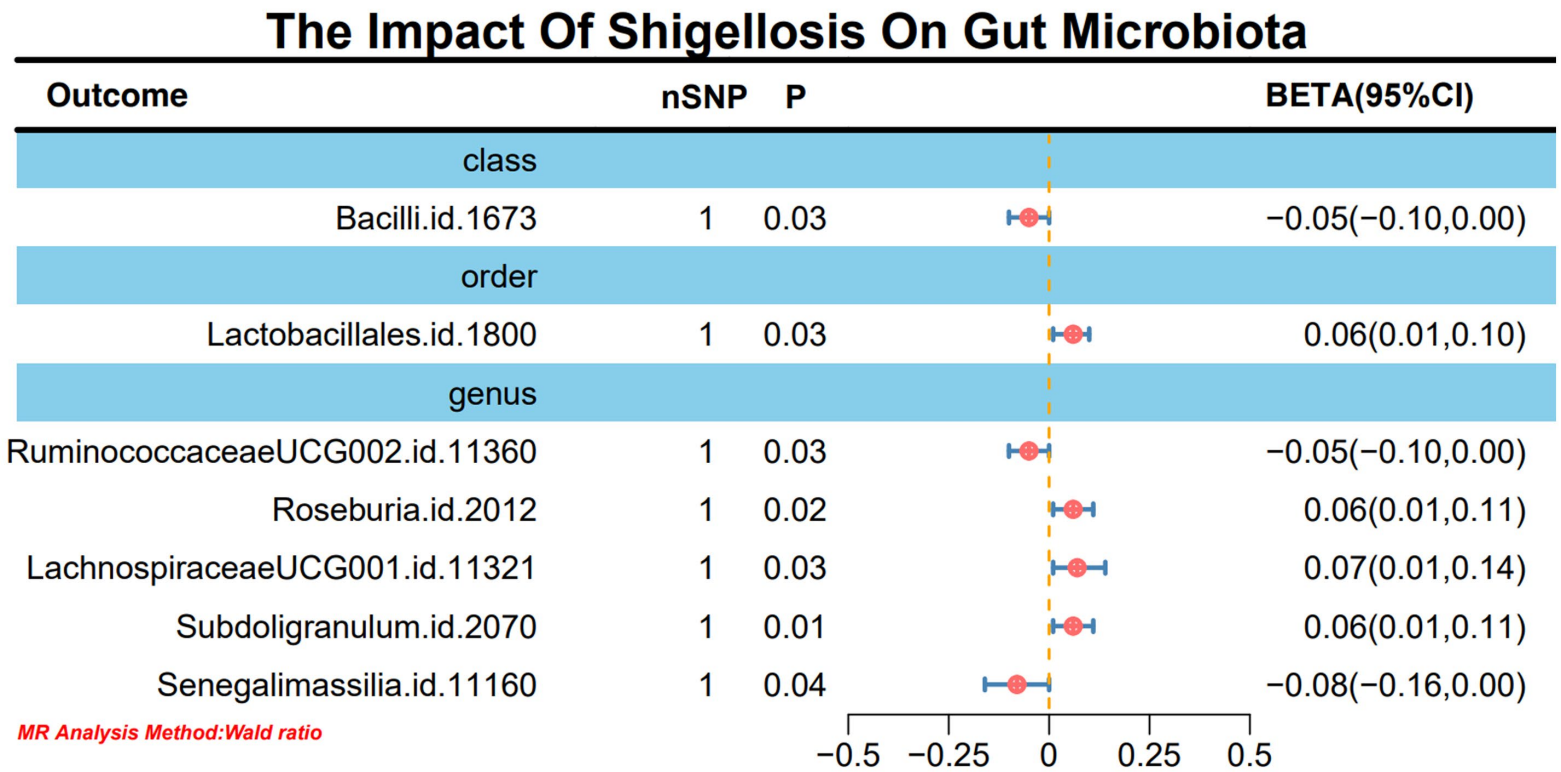
MR forest plot analysis delineates the causal impact of *Shigella* infection on the gut microbiota.

In light of the limited SNPs extracted from *Shigella* infection, a Wald ratio method was employed for MR analysis to scrutinize the potential associations between *Shigella* infection and the gut microbiota. The outcomes revealed noteworthy associations with various taxonomic entities. Specifically, class Bacilli (BETA: −0.05, 95% CI: −0.10–0.00, *p* = 0.02), order Lactobacillales (BETA: 0.06, 95% CI: 0.01–0.10, *p* = 0.03), genus *RuminococcaceaeUCG002* (BETA: −0.05, 95% CI: −0.01-0.00, *p* = 0.03), genus *Roseburia* (BETA: 0.06, 95% CI: 0.01–0.11, *p* = 0.02), genus *LachnospiraceaeUCG001* (BETA: 0.07, 95% CI: 0.01–0.14, *p* = 0.03), genus *Subdoligranulum* (BETA: 0.06, 95% CI: 0.01–0.11, *p* = 0.01), and genus *Senegalimassilia* (BETA: −0.08, 95% CI: −0.16-0.00, *p* = 0.04) demonstrated discernible effects (Figure 3). It is worth noting that these outcomes retained stability even after subjected to sensitivity analyses (Supplementary material 1-S4).

**Figure 4:**
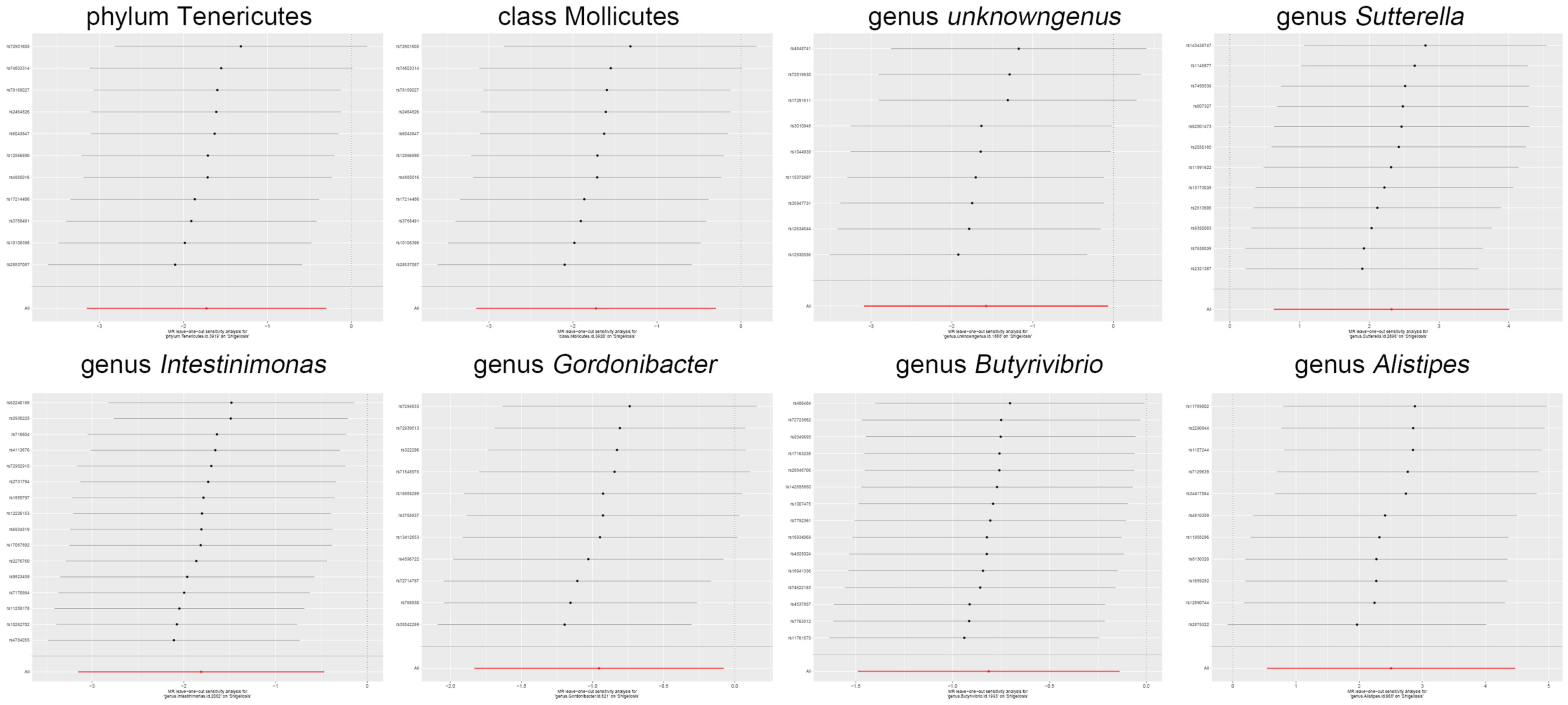
Leave-one-out analysis graphically depicts the causal associations between the gut microbiota and *Shigella* infection.

**Figure 5:**
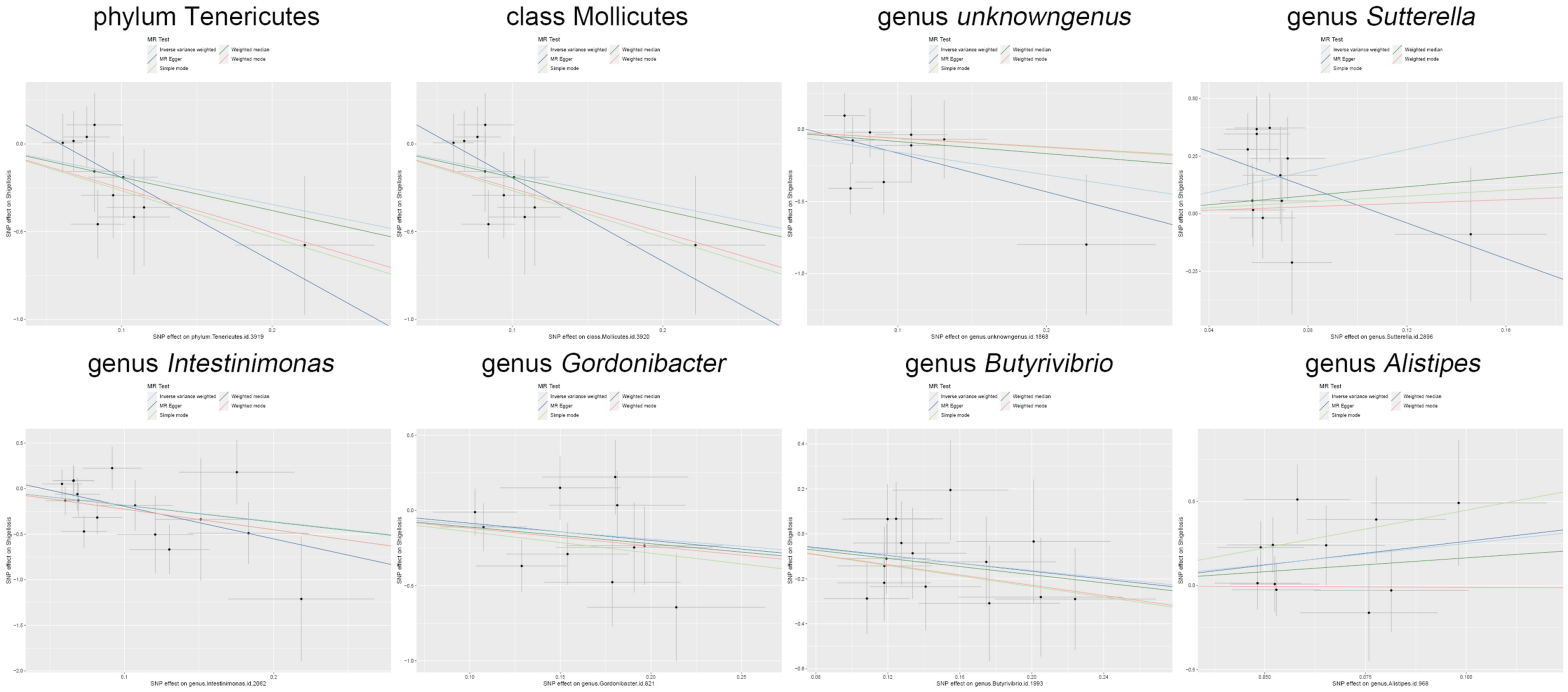
Scatter plot illustrates the causal associations between the gut microbiota and *Shigella* infection.

**Figure 6:**
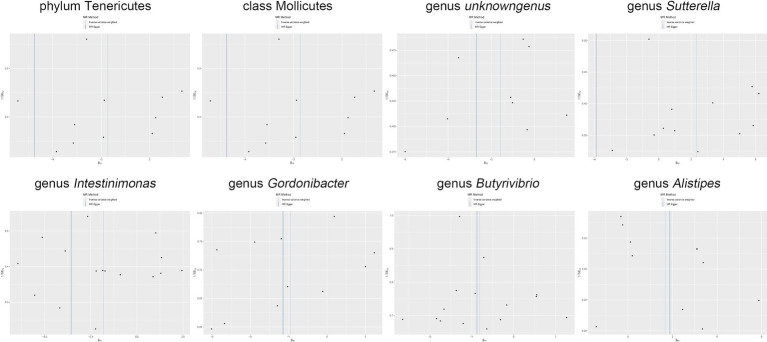
Funnel plot delineates the causal associations between the gut microbiota and *Shigella* infection.

Moreover, in our sensitivity analyses, stability of all outcomes was consistently upheld through the implementation of MR-Egger regression analysis and MR-PRESSO detection. Furthermore, the robustness of the MR analysis results was scrutinized using a leave-one-out analysis (depicted in Figure 4; Supplementary material 2), a scatter plot assessment (delineated in Figure 5; Supplementary material 3) and a funnel plot assessment (illustrated in Figure 6; Supplementary material 4). These assessments served to evaluate the resilience and reliability of our MR findings.

## Discussion

In our investigation, a bidirectional two-sample MR study was employed to elucidate the potential causal relationship between the gut microbiota and *Shigella* infection. Our findings unequivocally establish a reciprocal interaction between the gut microbiota and *Shigella* infection. Specifically, two microbial genera were identified as risk factors for *Shigella* infection, while six genera exhibited a protective role against the onset of *Shigella* infection. Importantly, in the outcomes of the reverse MR analysis, *Shigella* infection was shown to exert an impact on the composition of the gut microbiota, leading to alterations in four microbial genera.

Due to the predominant affliction site of *Shigella* infection within the gastrointestinal tract, coupled with the invasive nature of *Shigella* bacterial infiltration and resultant damage to intestinal epithelial cells, an indisputable and intricate correlation exists between *Shigella* infection and the gastrointestinal tract, as well as the gut microbiota (Baker and The, 2018). However, conventional research methodologies face formidable challenges in disentangling this relationship due to the myriad confounding factors. Consequently, we employed MR analysis, providing a robust framework to systematically investigate the causal relationship between *Shigella* infection and the gut microbiota.

In our investigation, we observed an inverse correlation between phylum Tenericutes, class Mollicutes, genus *Intestinimonas*, genus *Gordonibacter*, genus *Butyrivibrio*, and *Shigella* infection, while genus *Sutterella* and genus *Alistipes* exhibited a positive association with *Shigella* infection. Notably, phylum Tenericutes and class Mollicutes emerged as protective factors against *Shigella* infection, consistent with previous research indicating their negative correlation with inflammatory responses, particularly during the accumulation of C-reactive protein (CRP; Ren et al., 2023). CRP has been previously recognized as a marker of *Shigella* infection activity (Khan et al., 1995). Genus *Sutterella*, implicated in conditions such as autism, Down syndrome (Biagi et al., 2014), and inflammatory bowel disease (Lavelle et al., 2015), displayed a positive association with *Shigella* infection. In the human gastrointestinal tract, genus *Sutterella* exhibits varying abundance, with higher levels in the duodenum of healthy adults, decreasing toward the colon. Its correlation with the adhesion capacity of intestinal epithelial cells and intestinal permeability is highly significant, potentially fostering the occurrence of inflammatory reactions (Hiippala et al., 2016). Notably, the subtype *Sutterella wadsworthensis*, with strong adhesion capabilities to mucin and extracellular matrix proteins, induces the production of interleukin-8 by intestinal epithelial cells. Interleukin-8 and CRP serve as markers for bacterial infections (Franz et al., 1999), thereby establishing a close association with *Shigella* infection. Consequently, genus *Sutterella* may constitute a potential therapeutic target for *Shigella* infection. Genus *Intestinimonas* plays a pivotal role in promoting the metabolism of lysine into butyrate, a common anti-inflammatory short-chain fatty acid (Bui et al., 2020). On the other hand, genus *Gordonibacter*, associated with increased abundance in Crohn’s disease progression and closely linked to immune and inflammatory responses, aligns with our findings of an elevated correlation with *Shigella* infection and increased CRP levels (Salem et al., 2019). Similarly, the extensively studied butyrate-producing bacterium, genus *Butyrivibrio*, known to enhance intestinal mucosal barrier function and alleviate inflammation (Ye et al., 2021), exhibited a negative correlation with *Shigella* infection in our MR analysis. Conversely, genus *Alistipes* emerged as a risk factor in our study due to its close association with the activation pathway of interleukin-6, increasing the risk of inflammatory reactions in the host (Feng et al., 2015). In several animal experiments, it has also been clearly demonstrated that a decrease in the abundance of genus *Alistipes* occurs during the progression of infection with *Shigella flexneri*, which is consistent with the findings of our study analysis (Yang et al., 2020; Calvigioni et al., 2024). Consequently, these findings underscore the potential of these microbial taxa as biomarkers and therapeutic targets for *Shigella* infection, providing insights into the intricate interplay between the gut microbiota and the pathogenesis of *Shigella*-related disorders.

In the outcome of our reverse MR analysis, we infer that *Shigella* infection disrupts the equilibrium of the gut microbiota, leading to dysbiosis characterized by a decrease in the abundance of class Bacilli, genus *RuminococcaceaeUCG002*, and genus *Senegalimassilia*, coupled with a significant elevation in the abundance of order Lactobacillales, genus *Roseburia*, genus *LachnospiraceaeUCG001*, and genus *Subdoligranulum*. This observation aligns harmoniously with the findings from our forward MR analysis, thereby eliminating the possibility of reverse causation confounding our conclusions.

Ultimately, our research findings hold clear clinical significance. Whether it be *Shigella dysenteriae*, *Shigella flexneri*, *Shigella boydii*, or *Shigella sonnei*, their increasing infectiousness and the rampant misuse of antibiotics have contributed to a gradual surge in resistance, notably against frontline antibiotics such as cephalosporins (Matanza and Clements, 2023; Tsai et al., 2023). Given these circumstances, considering the protective role of gut microbiota, including bacteria like Genus *Butyrivibrio*, known for producing butyrate and reinforcing intestinal barriers, we regard them as potential interveners against the progression of the disease. Simultaneously, during the course of treating *Shigella* infections, intervening with collaborative pathogens such as genus *Sutterella* may yield significant therapeutic effects (Cai et al., 2022).

However, our study is not without limitations. Firstly, the data we utilized is derived from GWAS databases, and its availability is relatively constrained. Furthermore, the datasets pertaining to *Shigella* infection and gut microbiota predominantly originate from European populations. Secondly, our focus on the gut microbiota was primarily at the genus level, and we did not delve into specific bacterial species. Additionally, our examination of *Shigella* infection was comprehensive and did not involve subtyping, such as distinct analyses for *Shigella dysenteriae*, *Shigella flexneri*, *Shigella sonnei*, and *Shigella boydii*.

## Conclusion

Our MR analysis has substantiated the bidirectional causal relationship between the gut microbiota and *Shigella* infection. This study not only opens novel avenues for the prevention and treatment of *Shigella* infections but also furnishes new insights into unraveling the pathogenic mechanisms by which the gut microbiota influences the onset of *Shigella* infection.

## Data availability statement

The datasets presented in this study can be found in online repositories. The names of the repository/repositories and accession number(s) can be found in the article/Supplementary material.

## Ethics statement

Our analysis does not entail the collection of raw data; instead, we leverage previously published studies or publicly available GWAS summary data. Therefore, ethical approval is not required for our study.

## Author contributions

JP: Writing – original draft. KC: Writing – review & editing. GC: Formal analysis, Writing – review & editing, Data curation. LL: Data curation, Methodology, Writing – review & editing. LP: Conceptualization, Project administration, Writing – review & editing.
