# Supplementary figures and images for: Genetic evidence strengthens the bidirectional connection between gut microbiota and Shigella infection: insights from a two-sample Mendelian randomization study
Source: Front Microbiol. 2024 Mar 1;15:1361927. doi: 10.3389/fmicb.2024.1361927 (PMC10941758; doi:10.3389/fmicb.2024.1361927)

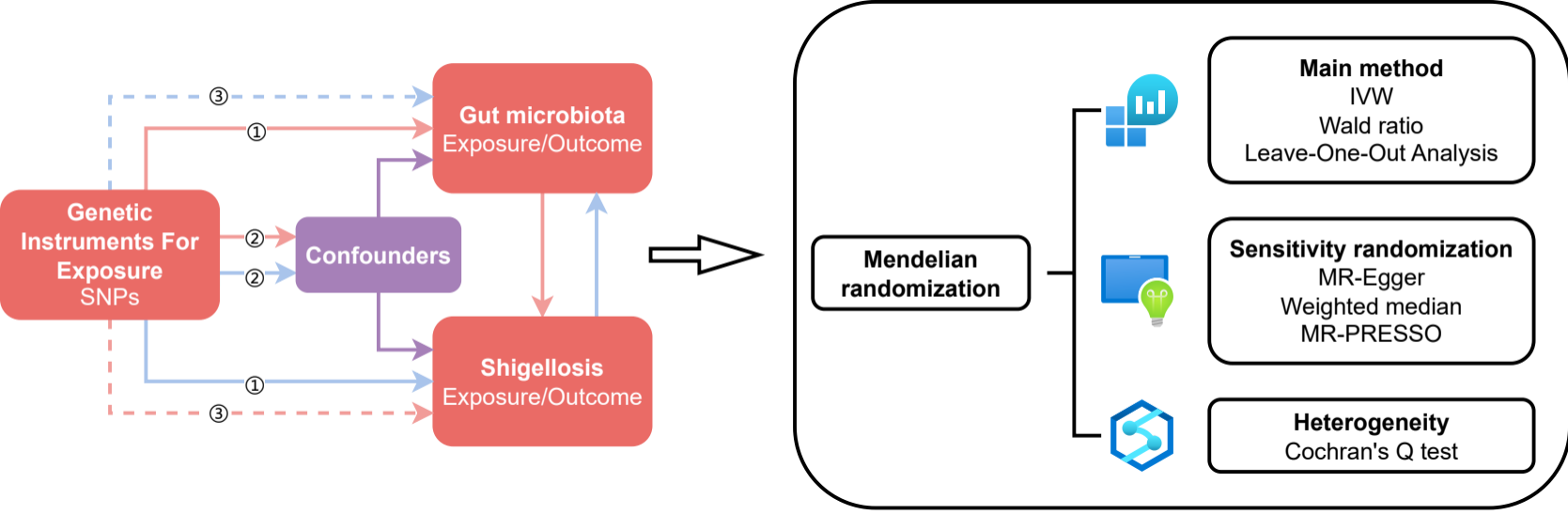

Supplement: Supplementary file 1 [file Data_Sheet_1.ZIP › Supplemenary Materials/Figure 1.pdf]

# The Effect Of Gut Microbiota On Shigellosis

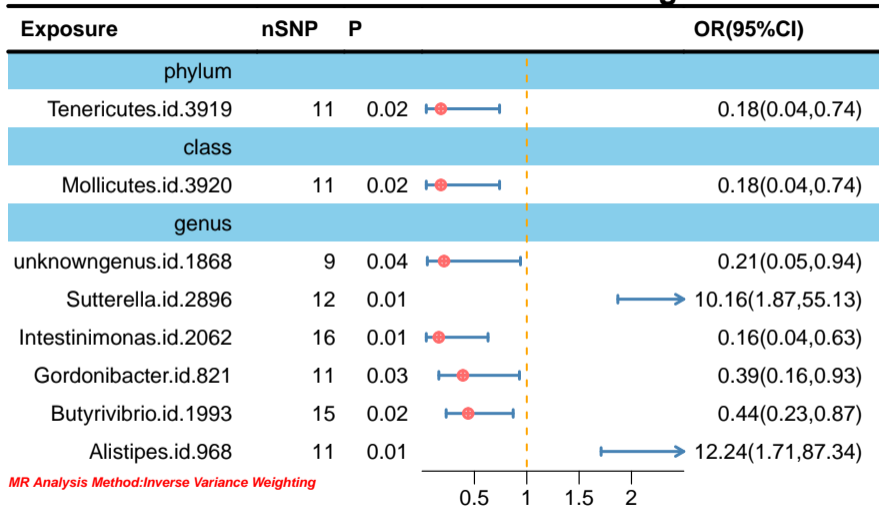

Supplement: Supplementary file 1 [file Data_Sheet_1.ZIP › Supplemenary Materials/Figure 2.pdf]

# The Impact Of Shigellosis On Gut Microbiota

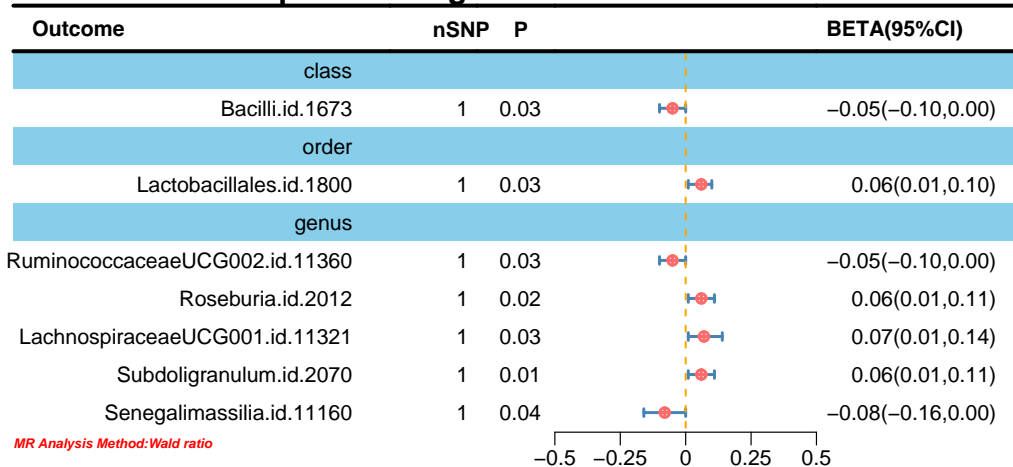

Supplement: Supplementary file 1 [file Data_Sheet_1.ZIP › Supplemenary Materials/Figure 3.pdf]

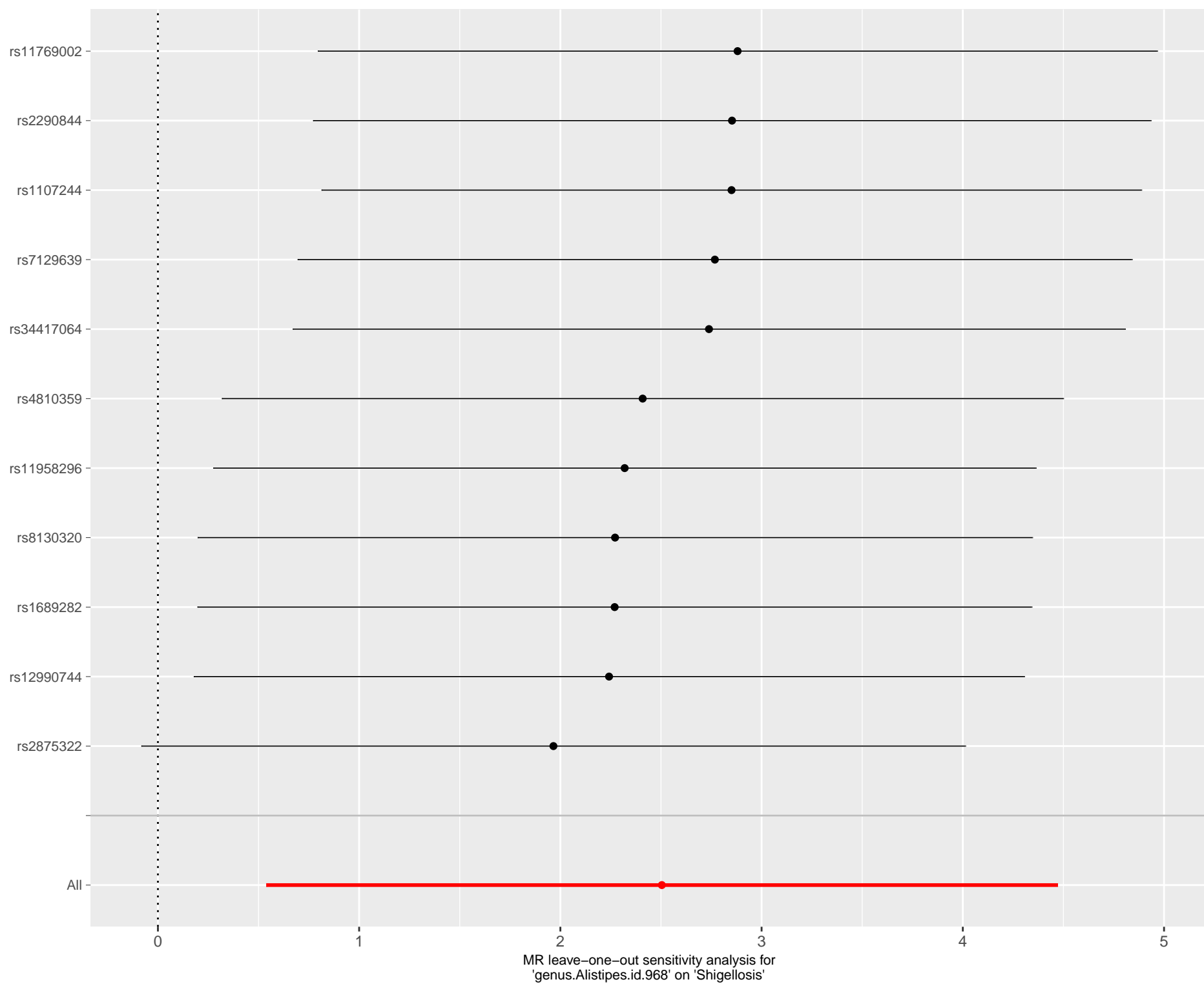

Supplement: Supplementary file 1 [file Data_Sheet_1.ZIP › Supplemenary Materials/Supplemenary Materials 2/Alistipes.pdf]

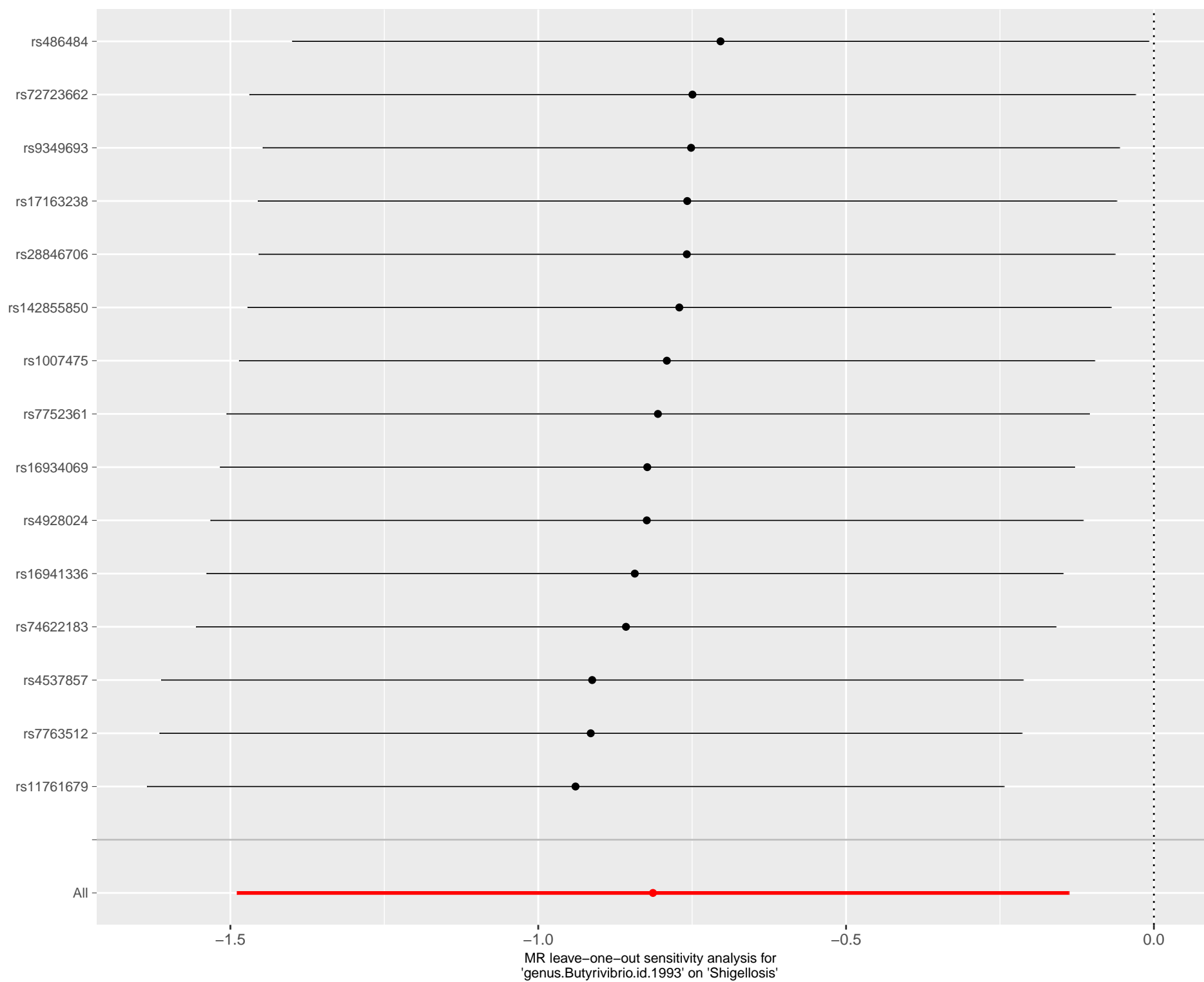

Supplement: Supplementary file 1 [file Data_Sheet_1.ZIP › Supplemenary Materials/Supplemenary Materials 2/Butyrivibrio.pdf]

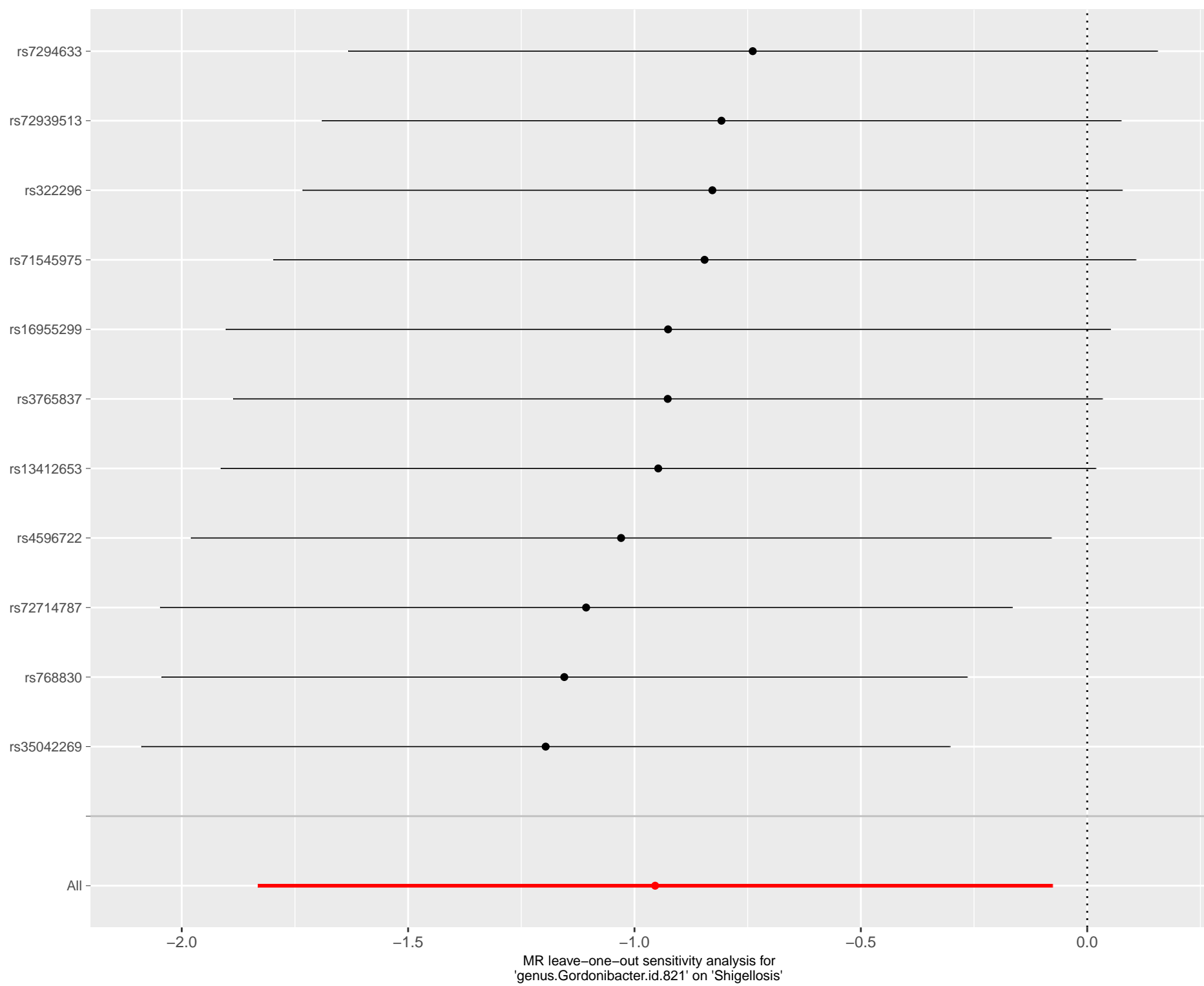

Supplement: Supplementary file 1 [file Data_Sheet_1.ZIP › Supplemenary Materials/Supplemenary Materials 2/Gordonibacter.pdf]

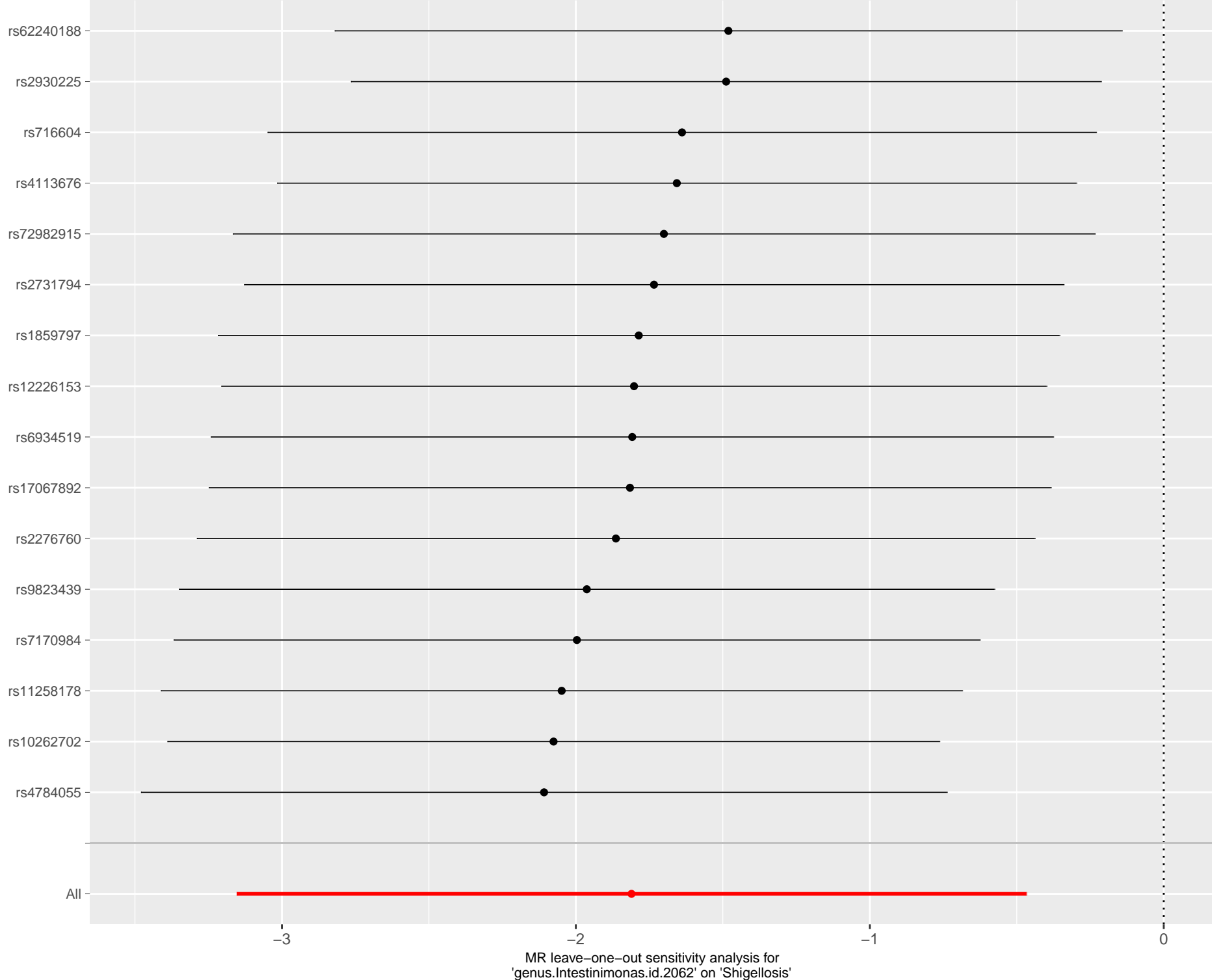

Supplement: Supplementary file 1 [file Data_Sheet_1.ZIP › Supplemenary Materials/Supplemenary Materials 2/Intestinimonas.pdf]

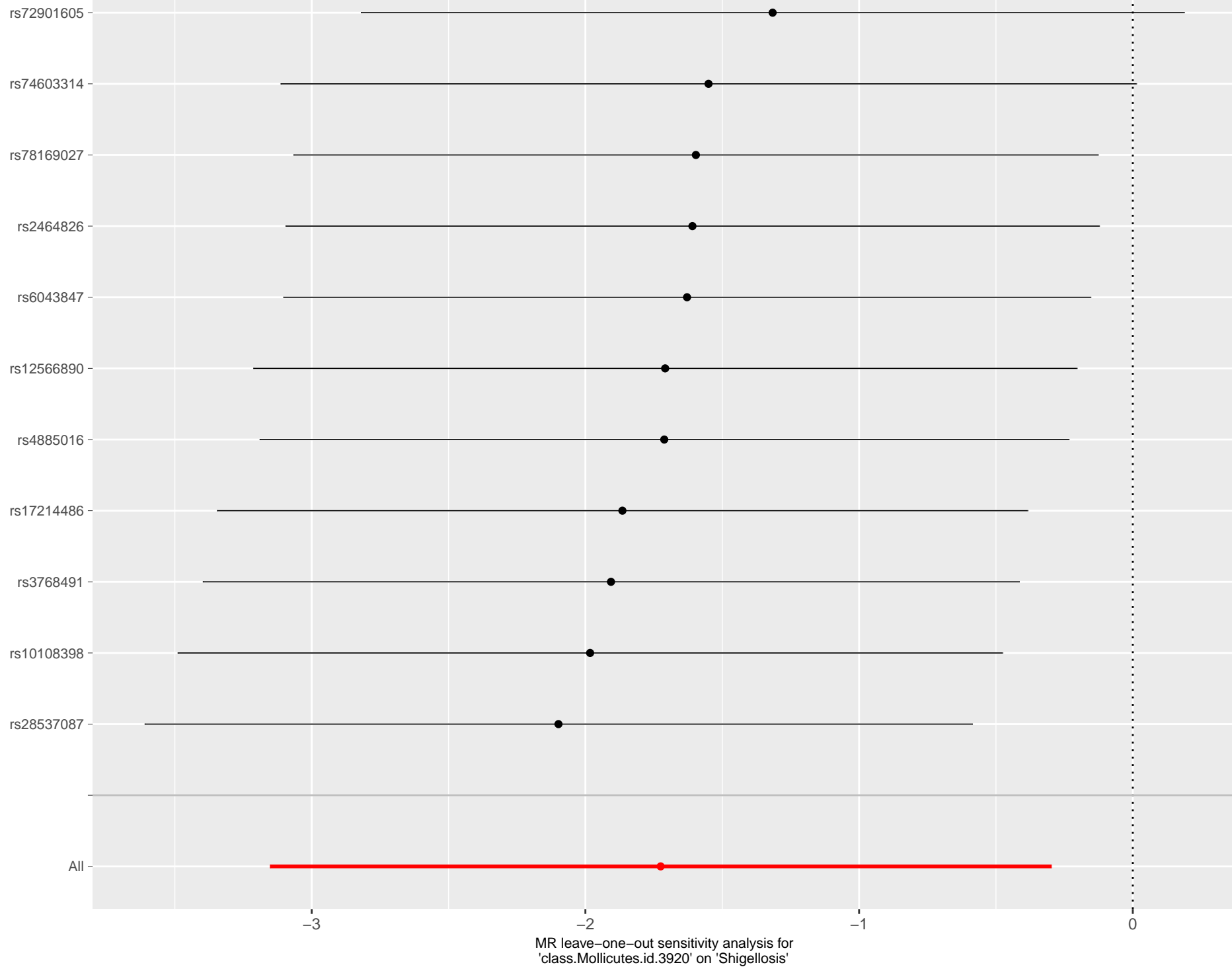

Supplement: Supplementary file 1 [file Data_Sheet_1.ZIP › Supplemenary Materials/Supplemenary Materials 2/Mollicutes.pdf]

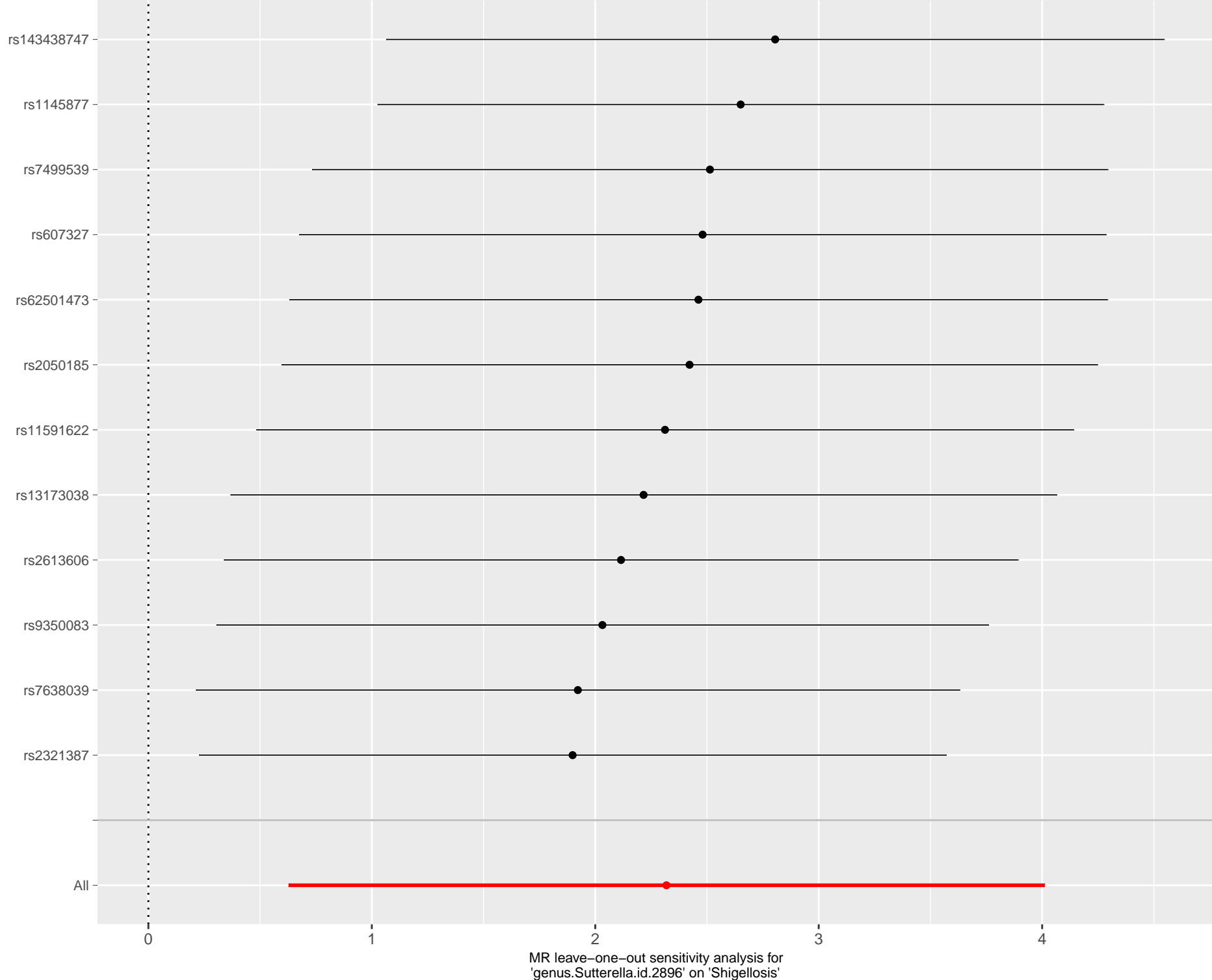

Supplement: Supplementary file 1 [file Data_Sheet_1.ZIP › Supplemenary Materials/Supplemenary Materials 2/Sutterella.pdf]

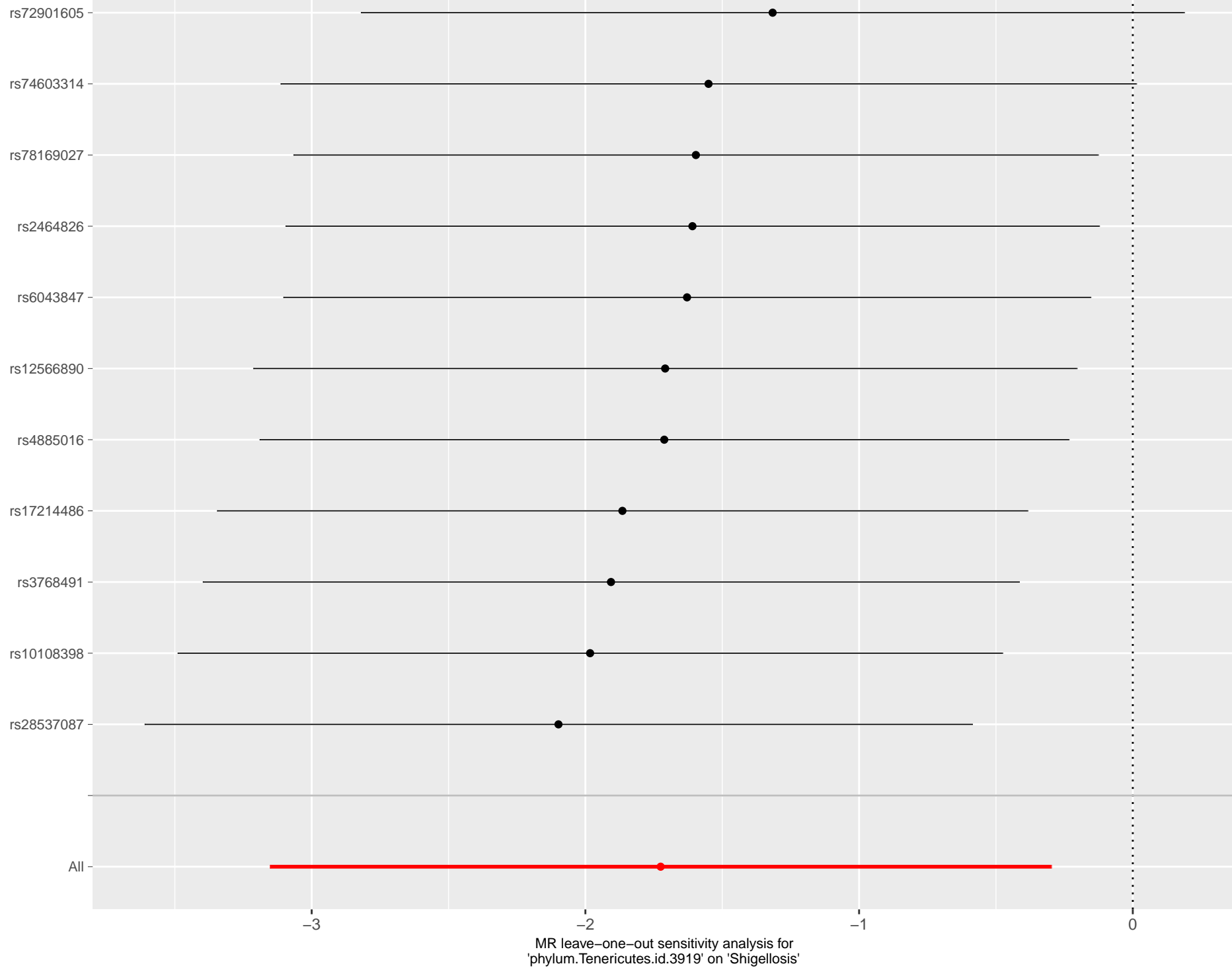

Supplement: Supplementary file 1 [file Data_Sheet_1.ZIP › Supplemenary Materials/Supplemenary Materials 2/Tenericutes.pdf]

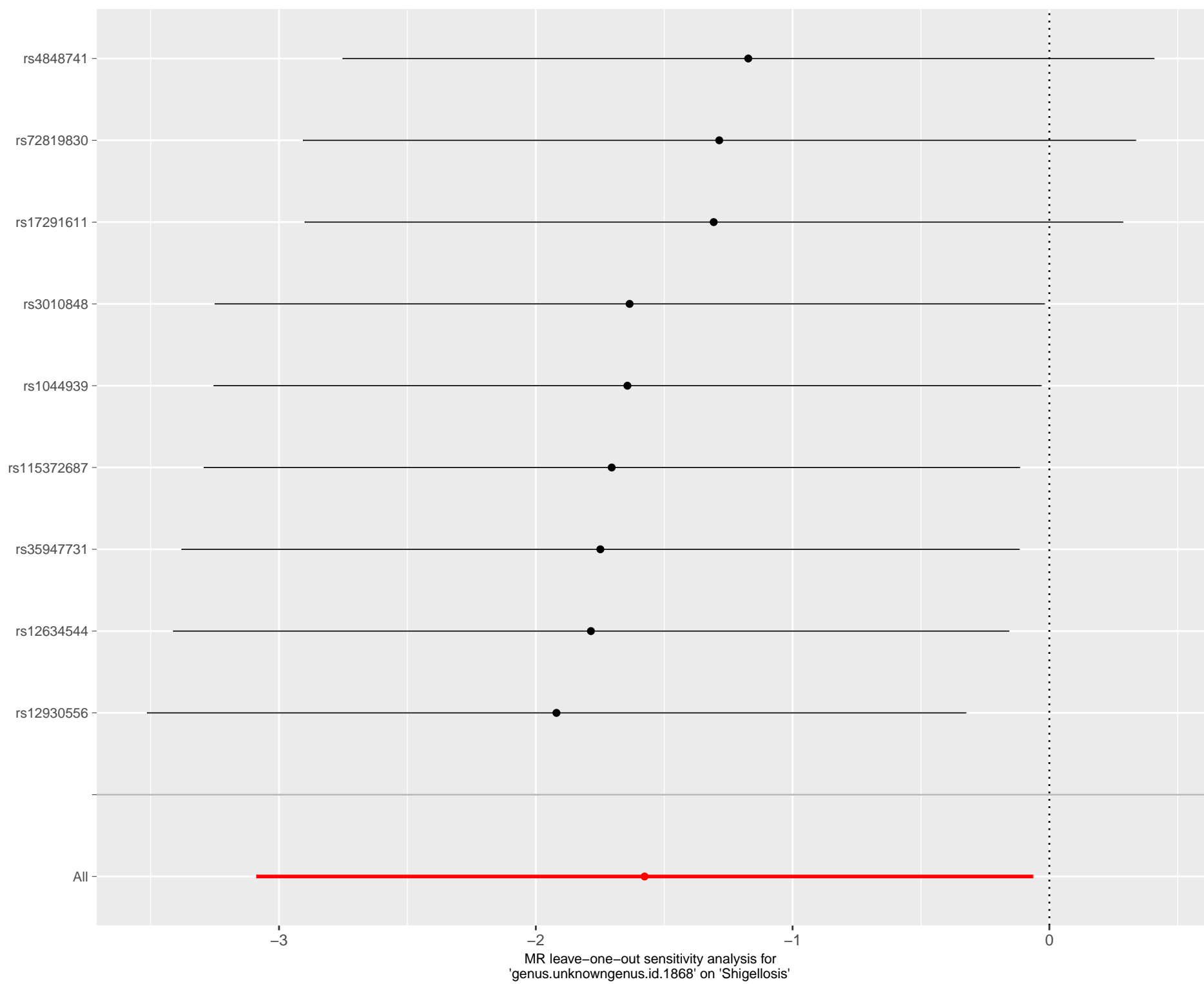

Supplement: Supplementary file 1 [file Data_Sheet_1.ZIP › Supplemenary Materials/Supplemenary Materials 2/unknowngenus.pdf]

# MR Test

- Inverse variance weighted
- MR Egger
- Simple mode
- Weighted median
- Weighted mode

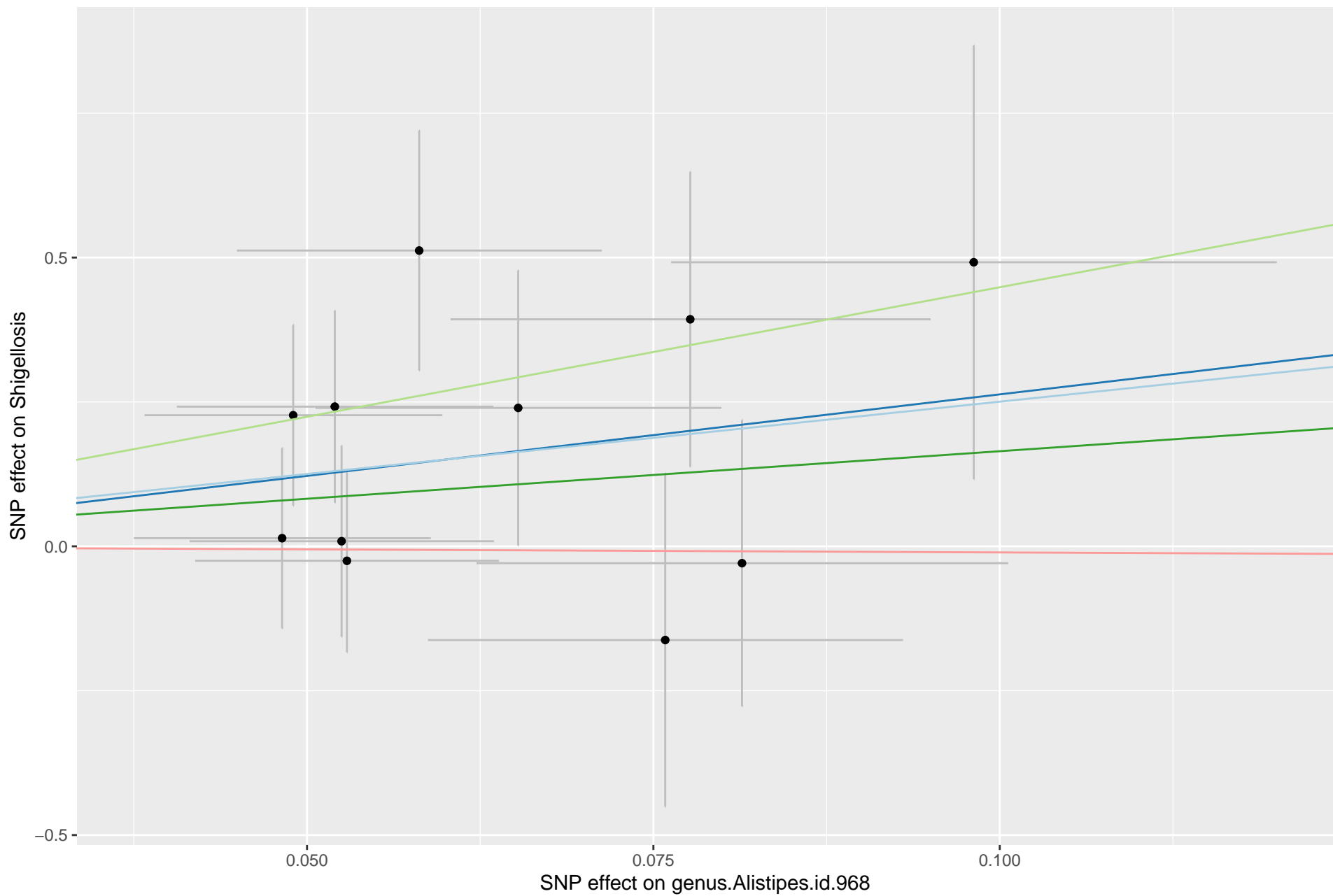

Supplement: Supplementary file 1 [file Data_Sheet_1.ZIP › Supplemenary Materials/Supplemenary Materials 3/Alistipes.pdf]

# MR Test

- Inverse variance weighted
- MR Egger
- Simple mode
- Weighted median
- Weighted mode

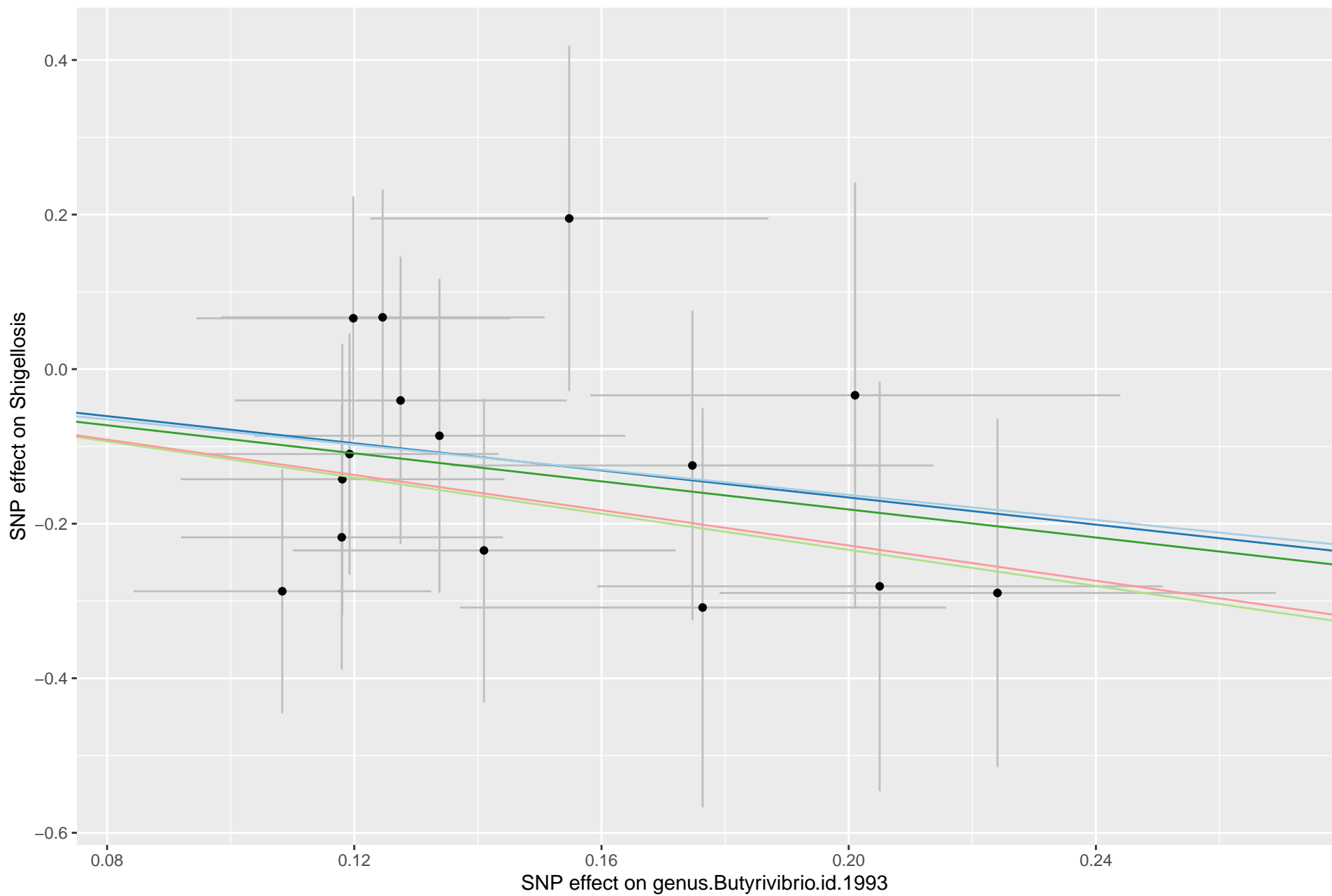

Supplement: Supplementary file 1 [file Data_Sheet_1.ZIP › Supplemenary Materials/Supplemenary Materials 3/Butyrivibrio.pdf]

# MR Test

- Inverse variance weighted
- MR Egger
- Simple mode
- Weighted median
- Weighted mode

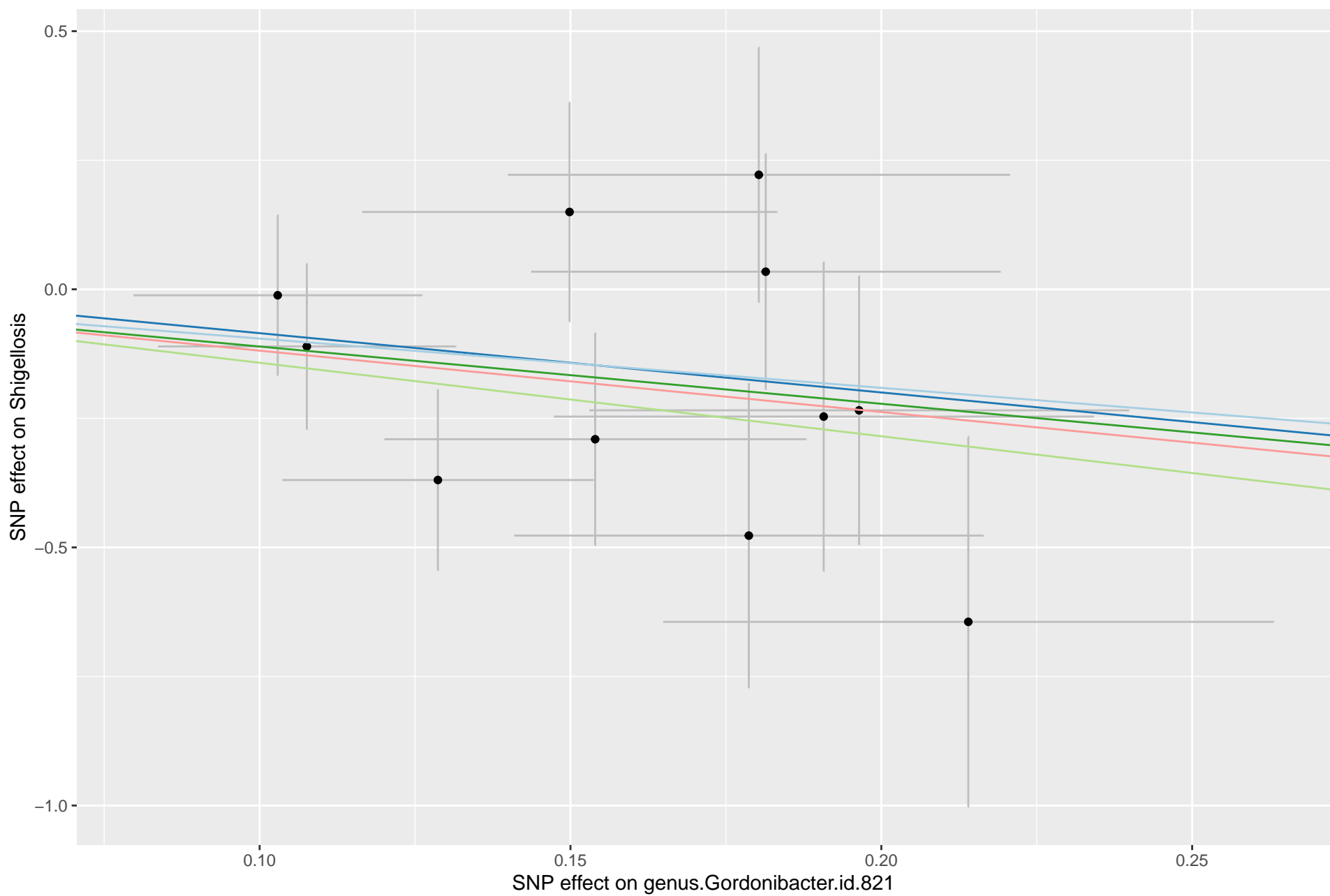

Supplement: Supplementary file 1 [file Data_Sheet_1.ZIP › Supplemenary Materials/Supplemenary Materials 3/Gordonibacter.pdf]

# MR Test

- Inverse variance weighted
- MR Egger
- Simple mode
- Weighted median
- Weighted mode

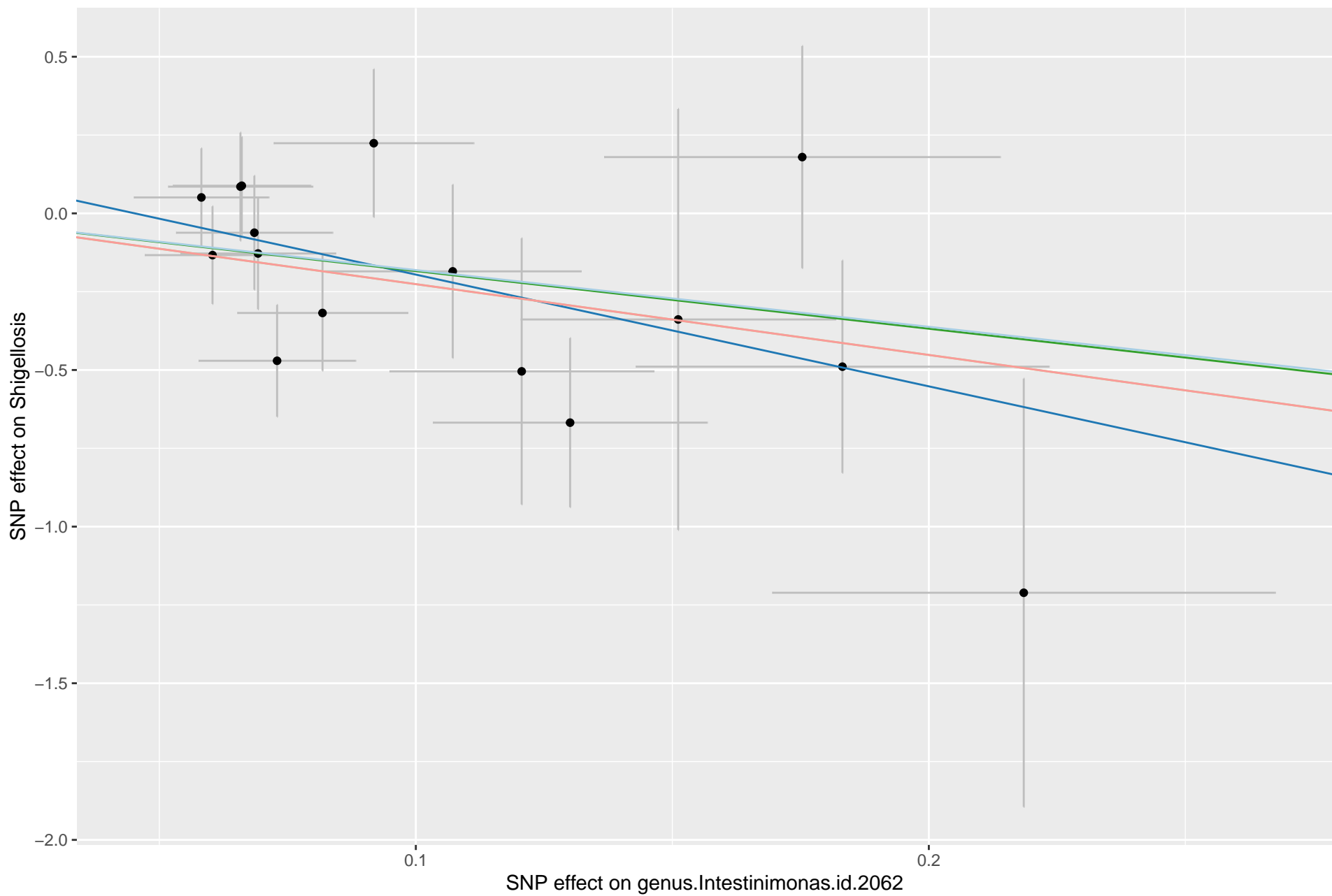

Supplement: Supplementary file 1 [file Data_Sheet_1.ZIP › Supplemenary Materials/Supplemenary Materials 3/Intestinimonas.pdf]

# MR Test

- Inverse variance weighted
- MR Egger
- Simple mode
- Weighted median
- Weighted mode

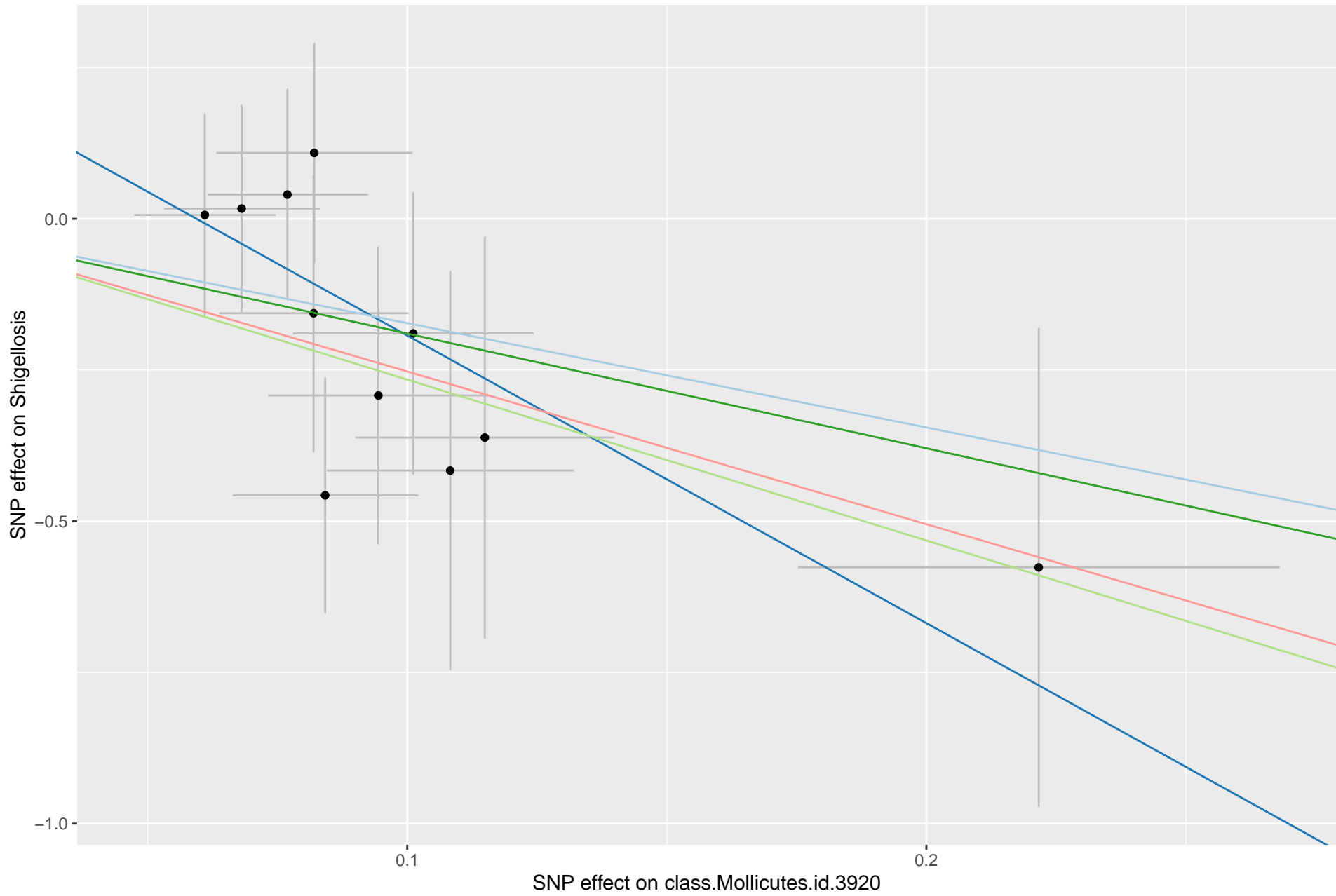

Supplement: Supplementary file 1 [file Data_Sheet_1.ZIP › Supplemenary Materials/Supplemenary Materials 3/Mollicutes.pdf]

# MR Test

- Inverse variance weighted
- MR Egger
- Simple mode
- Weighted median
- Weighted mode

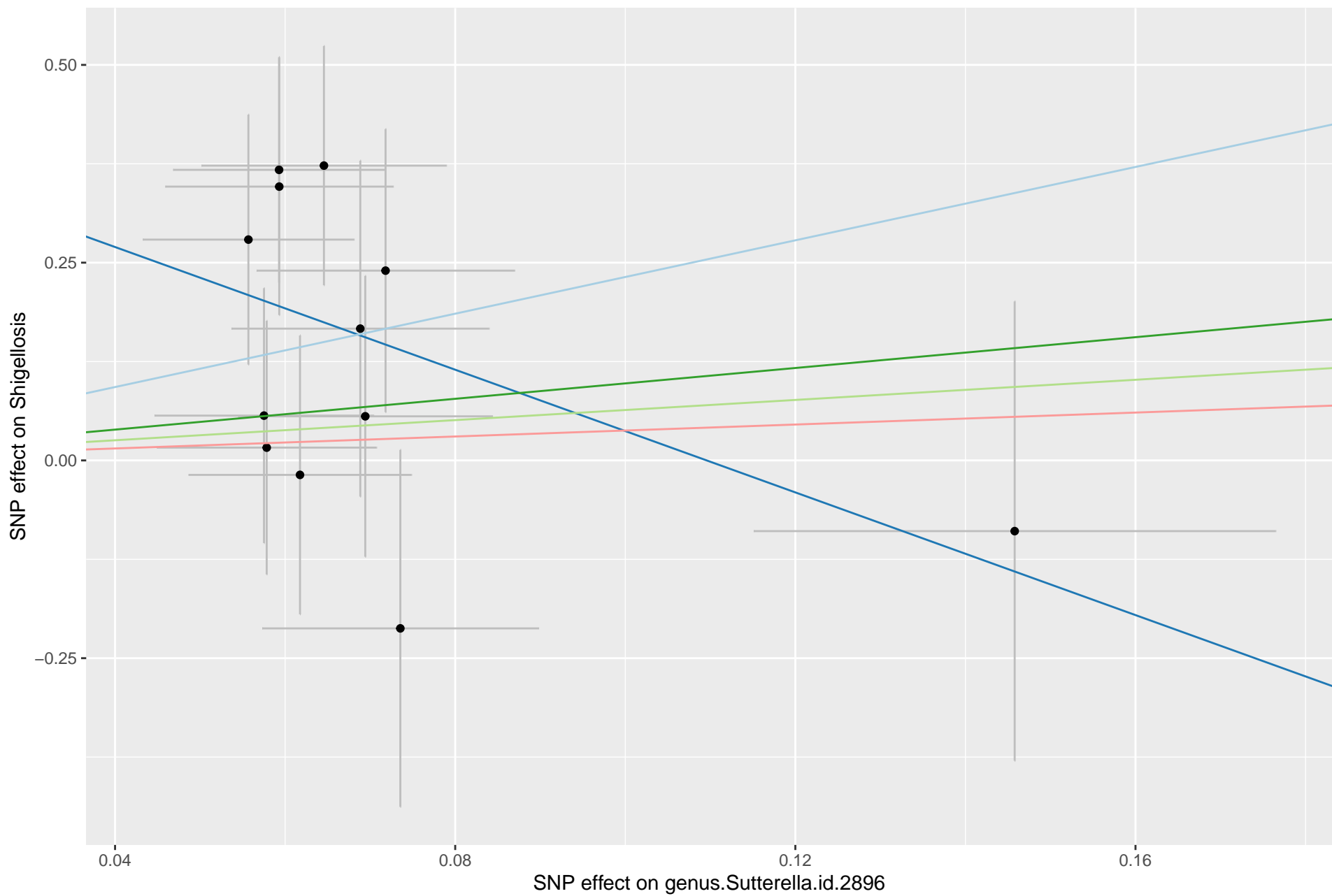

Supplement: Supplementary file 1 [file Data_Sheet_1.ZIP › Supplemenary Materials/Supplemenary Materials 3/Sutterella.pdf]

# MR Test

- Inverse variance weighted
- MR Egger
- Simple mode
- Weighted median
- Weighted mode

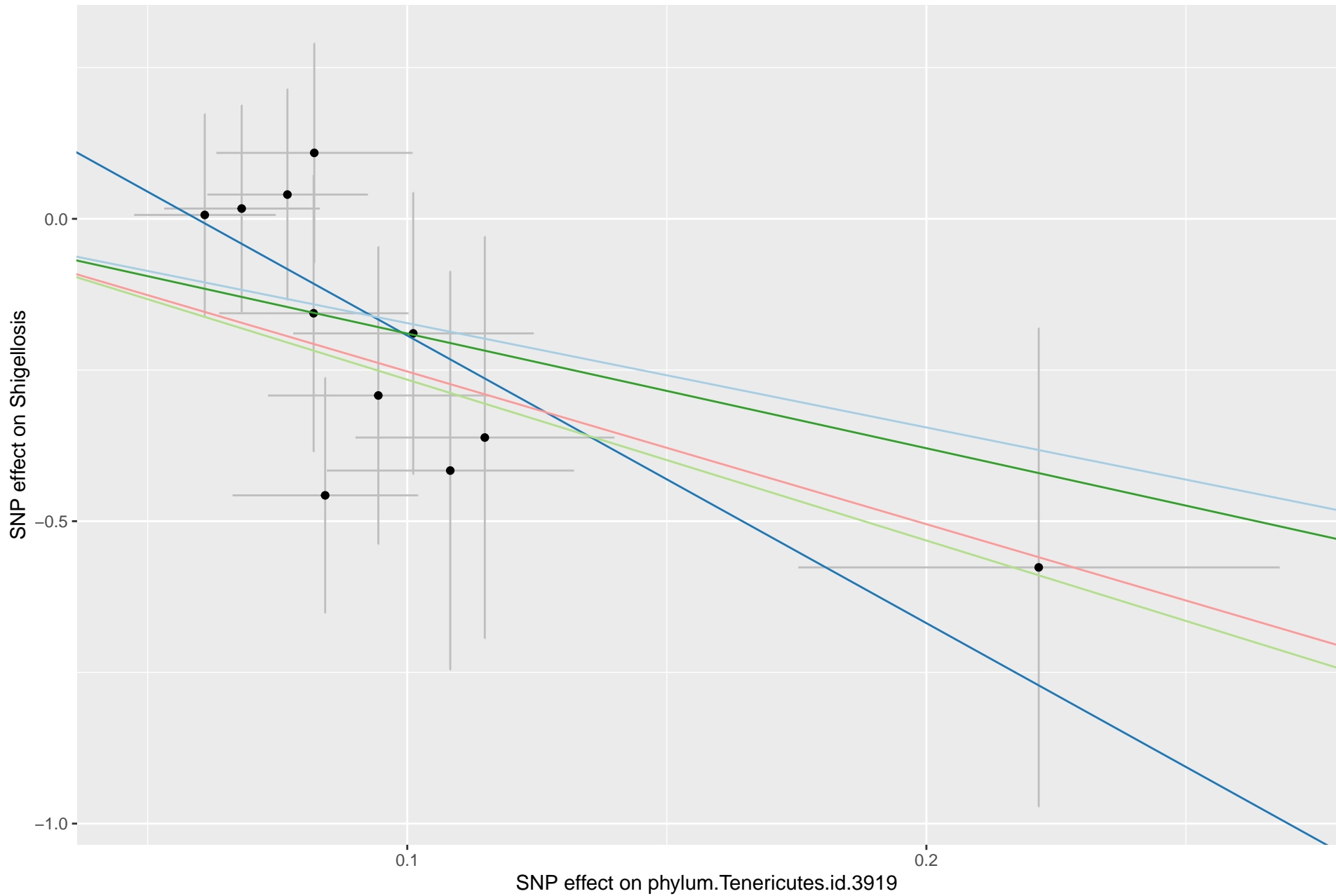

Supplement: Supplementary file 1 [file Data_Sheet_1.ZIP › Supplemenary Materials/Supplemenary Materials 3/Tenericutes .pdf]

# MR Test

- Inverse variance weighted
- MR Egger
- Simple mode
- Weighted median
- Weighted mode

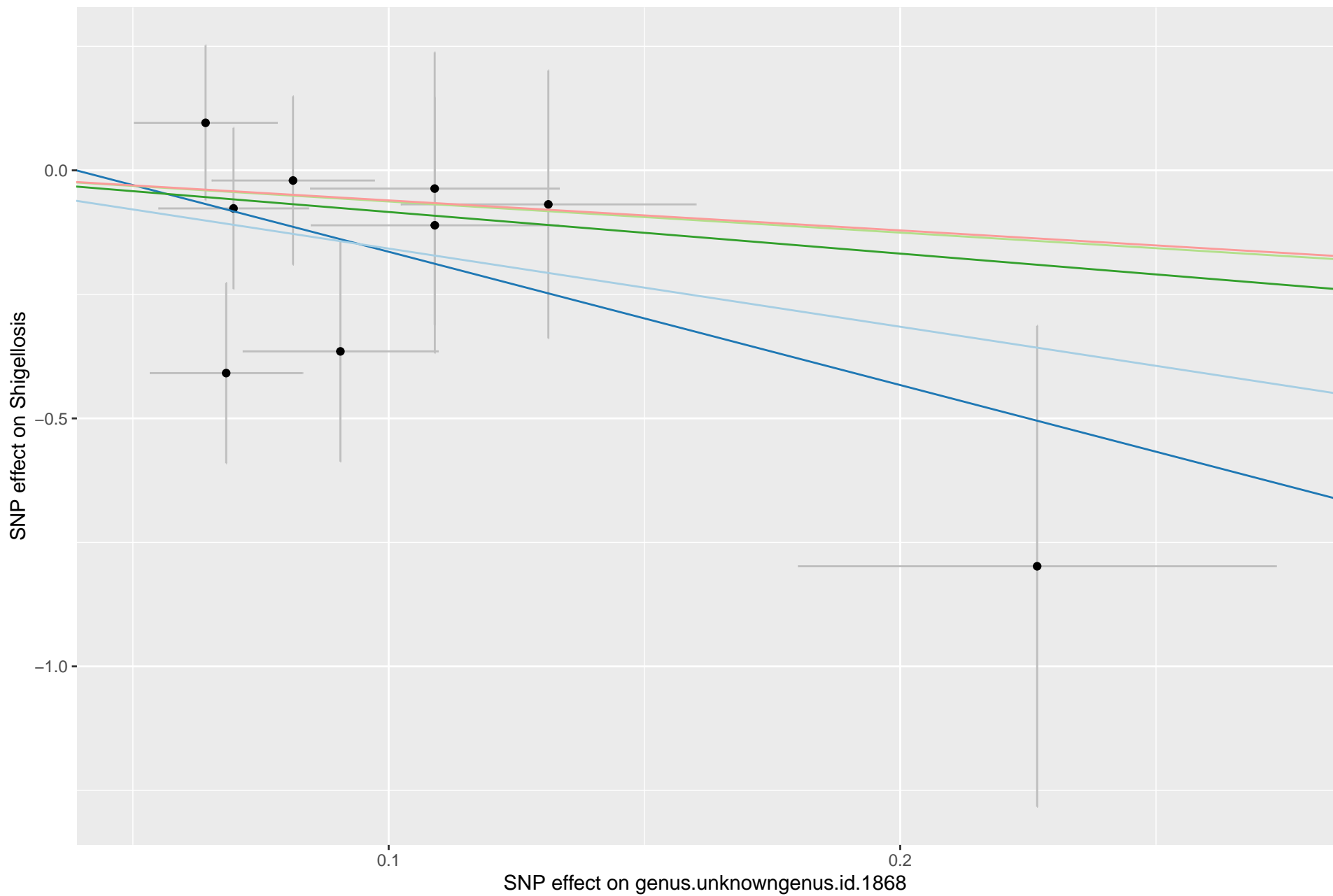

Supplement: Supplementary file 1 [file Data_Sheet_1.ZIP › Supplemenary Materials/Supplemenary Materials 3/unknowngenus.pdf]

# MR Method

- Inverse variance weighted
- MR Egger

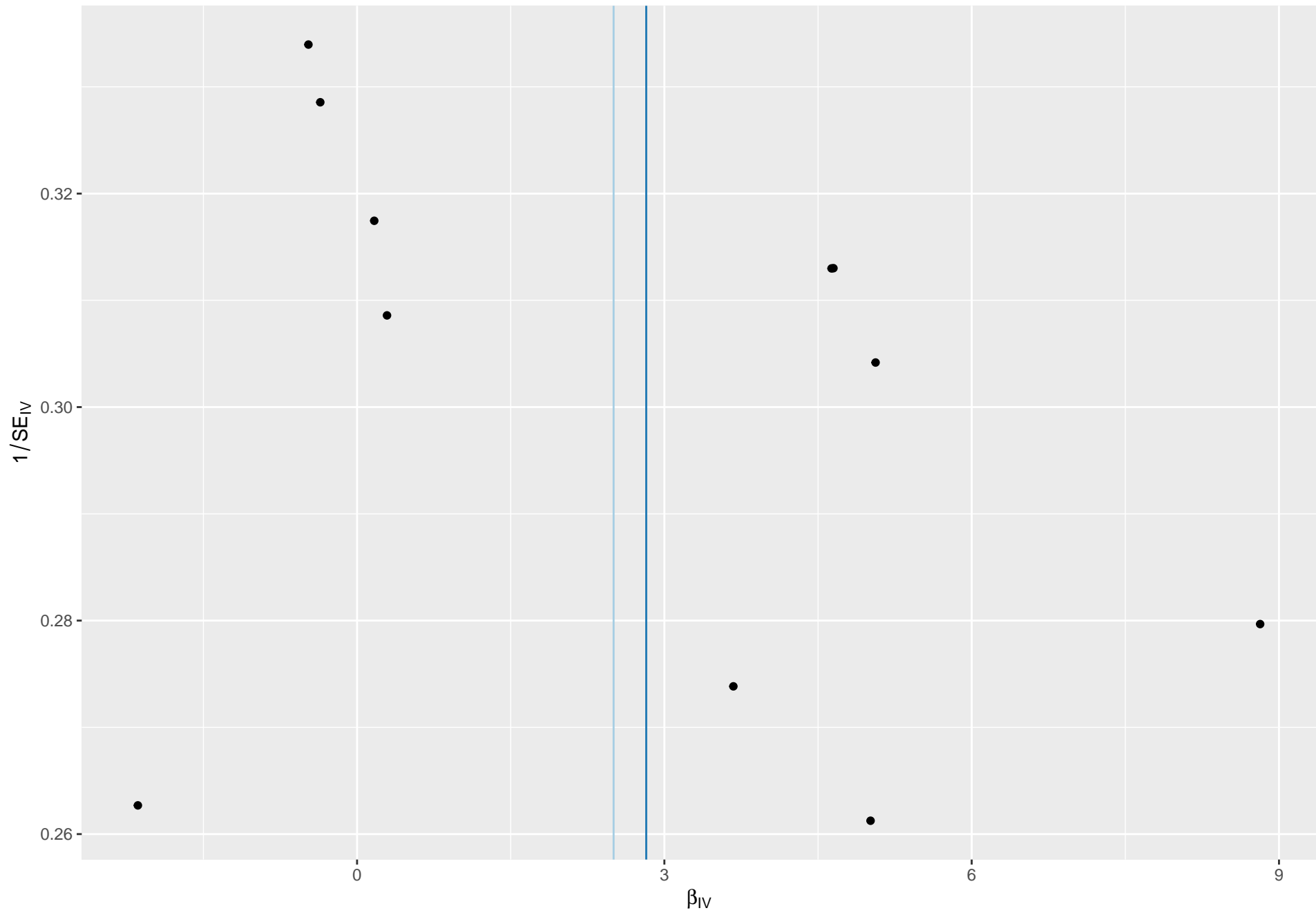

Supplement: Supplementary file 1 [file Data_Sheet_1.ZIP › Supplemenary Materials/Supplemenary Materials 4/Alistipes.pdf]

# MR Method

- Inverse variance weighted
- MR Egger

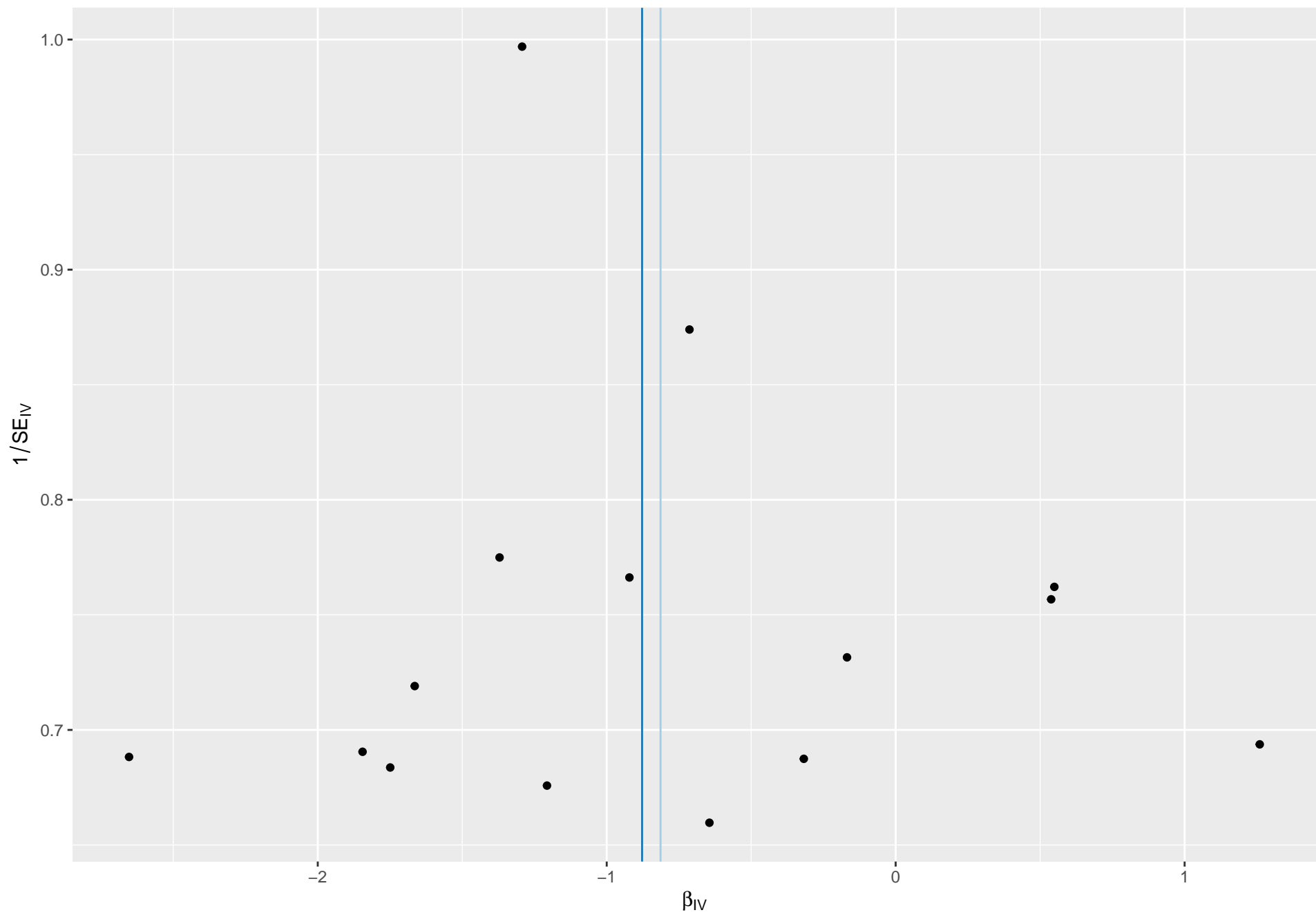

Supplement: Supplementary file 1 [file Data_Sheet_1.ZIP › Supplemenary Materials/Supplemenary Materials 4/Butyrivibrio.pdf]

# MR Method

- Inverse variance weighted
- MR Egger

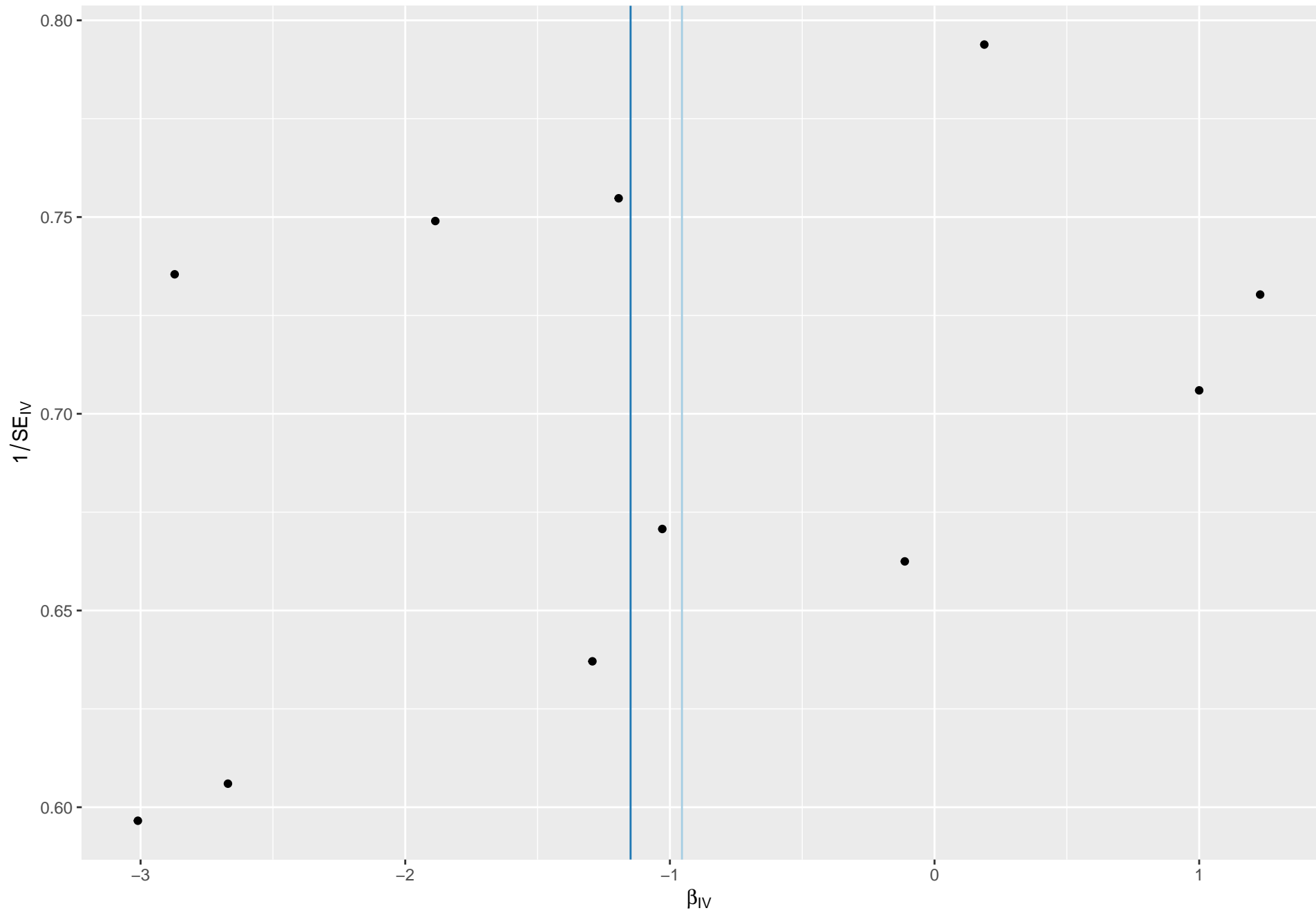

Supplement: Supplementary file 1 [file Data_Sheet_1.ZIP › Supplemenary Materials/Supplemenary Materials 4/Gordonibacter.pdf]

# MR Method

- Inverse variance weighted
- MR Egger

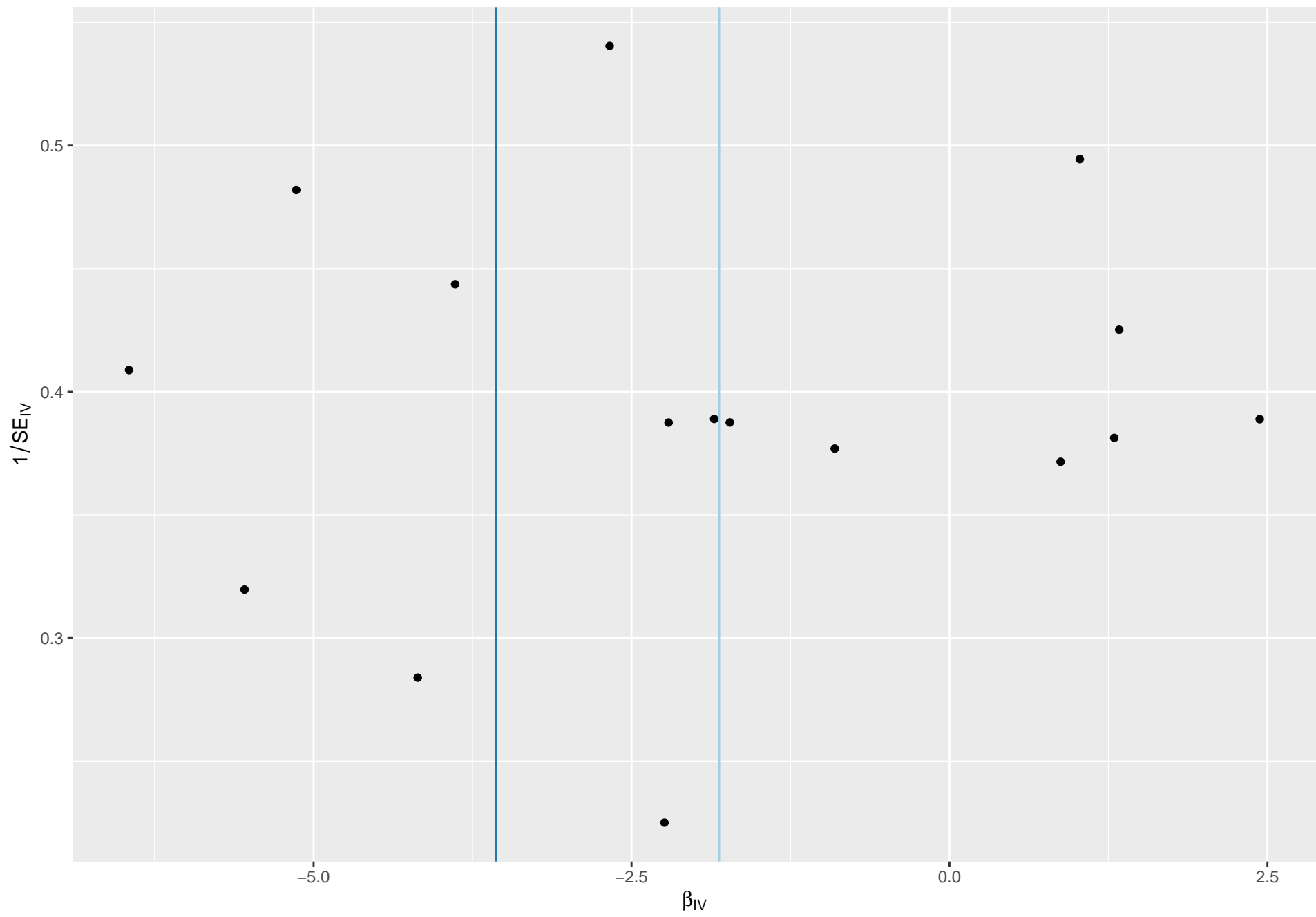

Supplement: Supplementary file 1 [file Data_Sheet_1.ZIP › Supplemenary Materials/Supplemenary Materials 4/Intestinimonas.pdf]

# MR Method

- Inverse variance weighted
- MR Egger

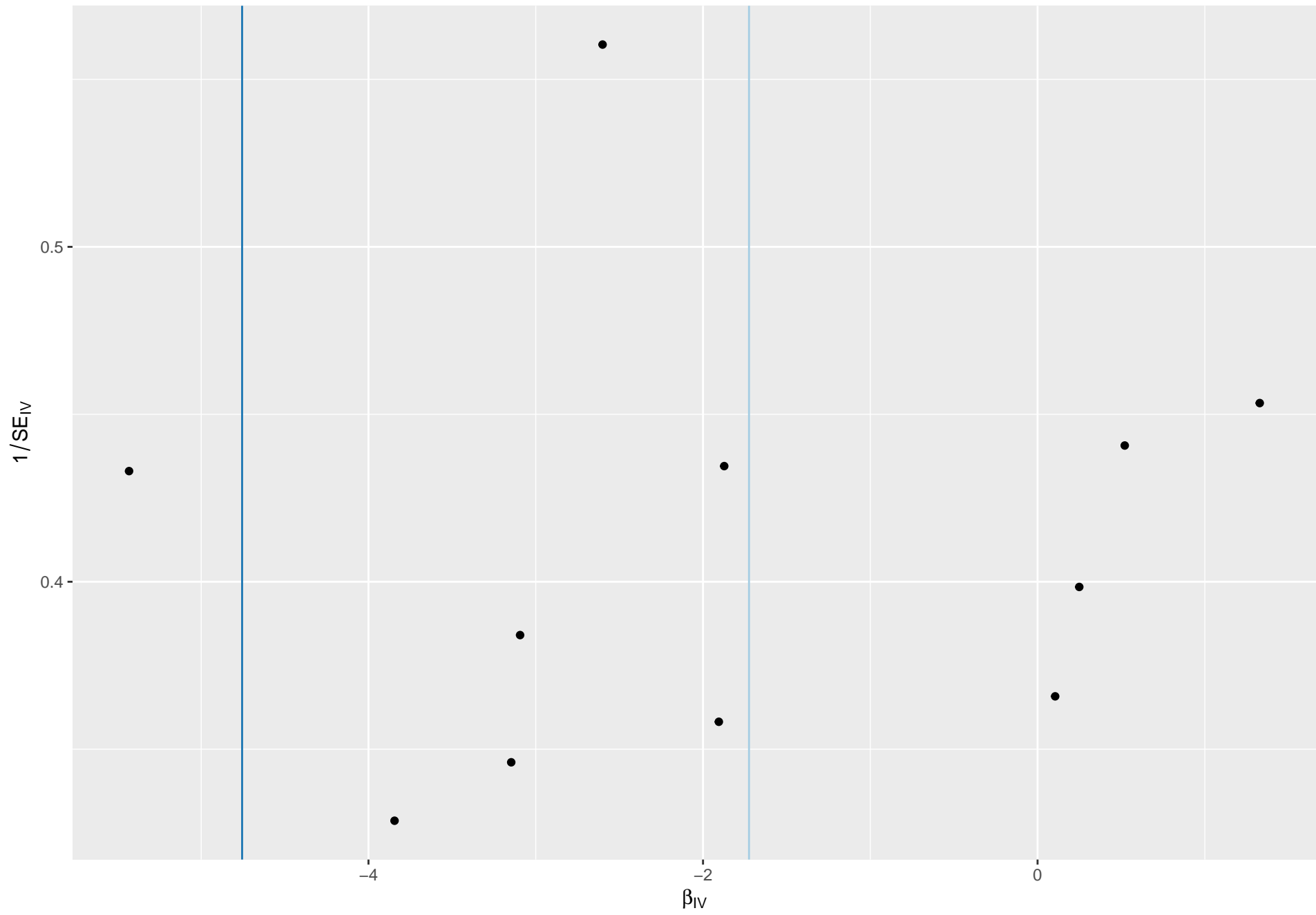

Supplement: Supplementary file 1 [file Data_Sheet_1.ZIP › Supplemenary Materials/Supplemenary Materials 4/Mollicutes.pdf]

# MR Method

- Inverse variance weighted
- MR Egger

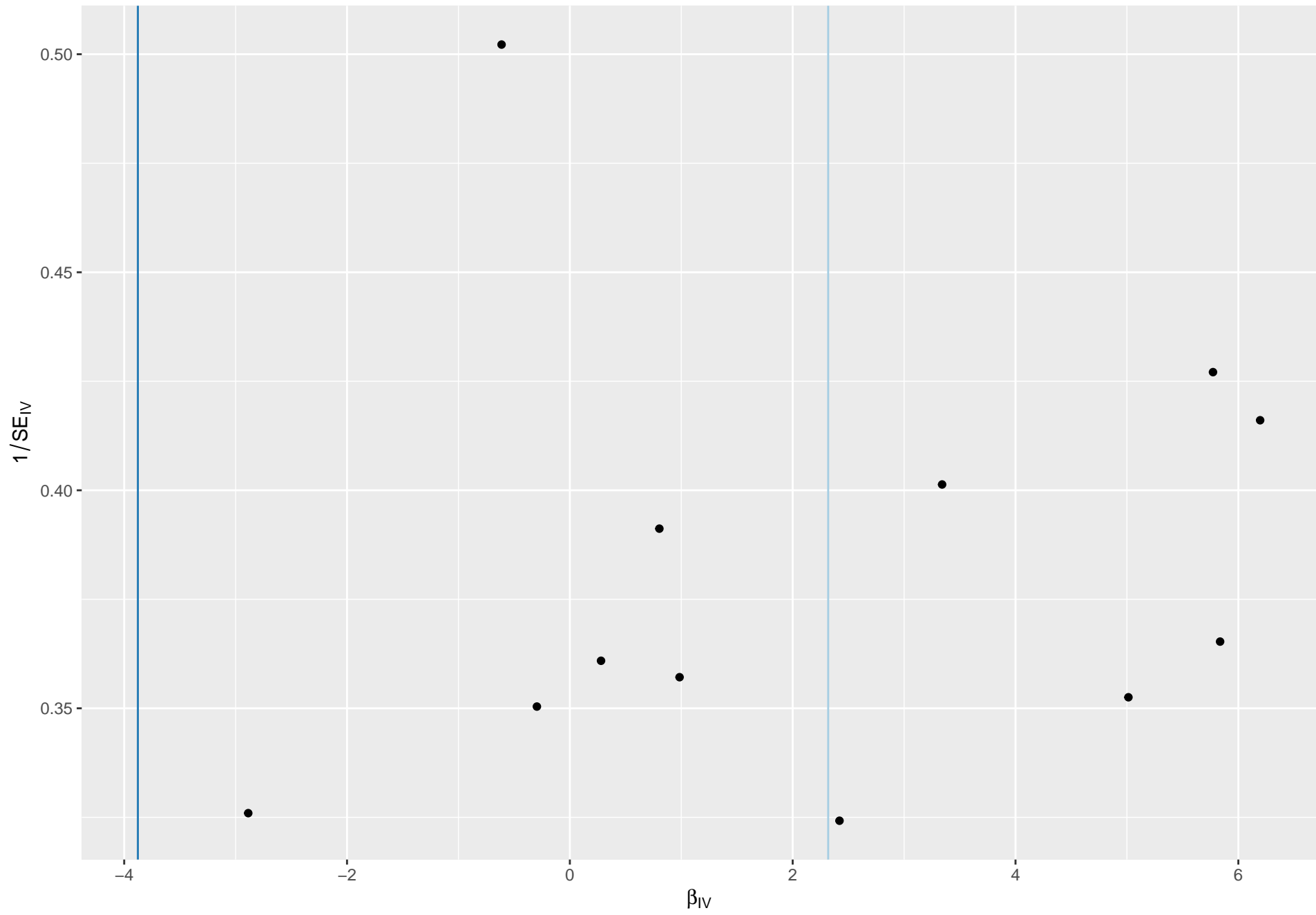

Supplement: Supplementary file 1 [file Data_Sheet_1.ZIP › Supplemenary Materials/Supplemenary Materials 4/Sutterella.pdf]

# MR Method

- Inverse variance weighted
- MR Egger

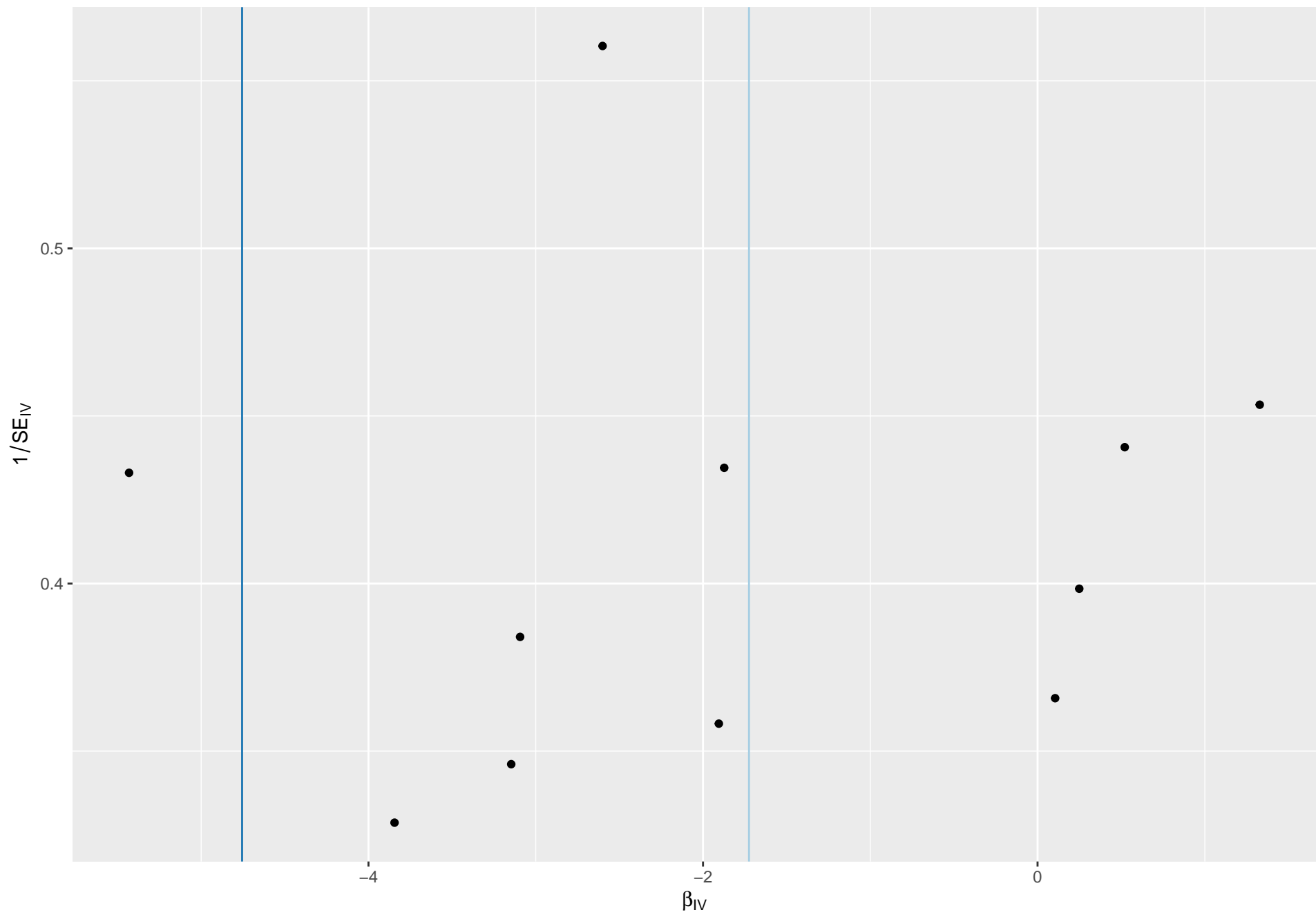

Supplement: Supplementary file 1 [file Data_Sheet_1.ZIP › Supplemenary Materials/Supplemenary Materials 4/Tenericutes.pdf]

# MR Method

- Inverse variance weighted
- MR Egger

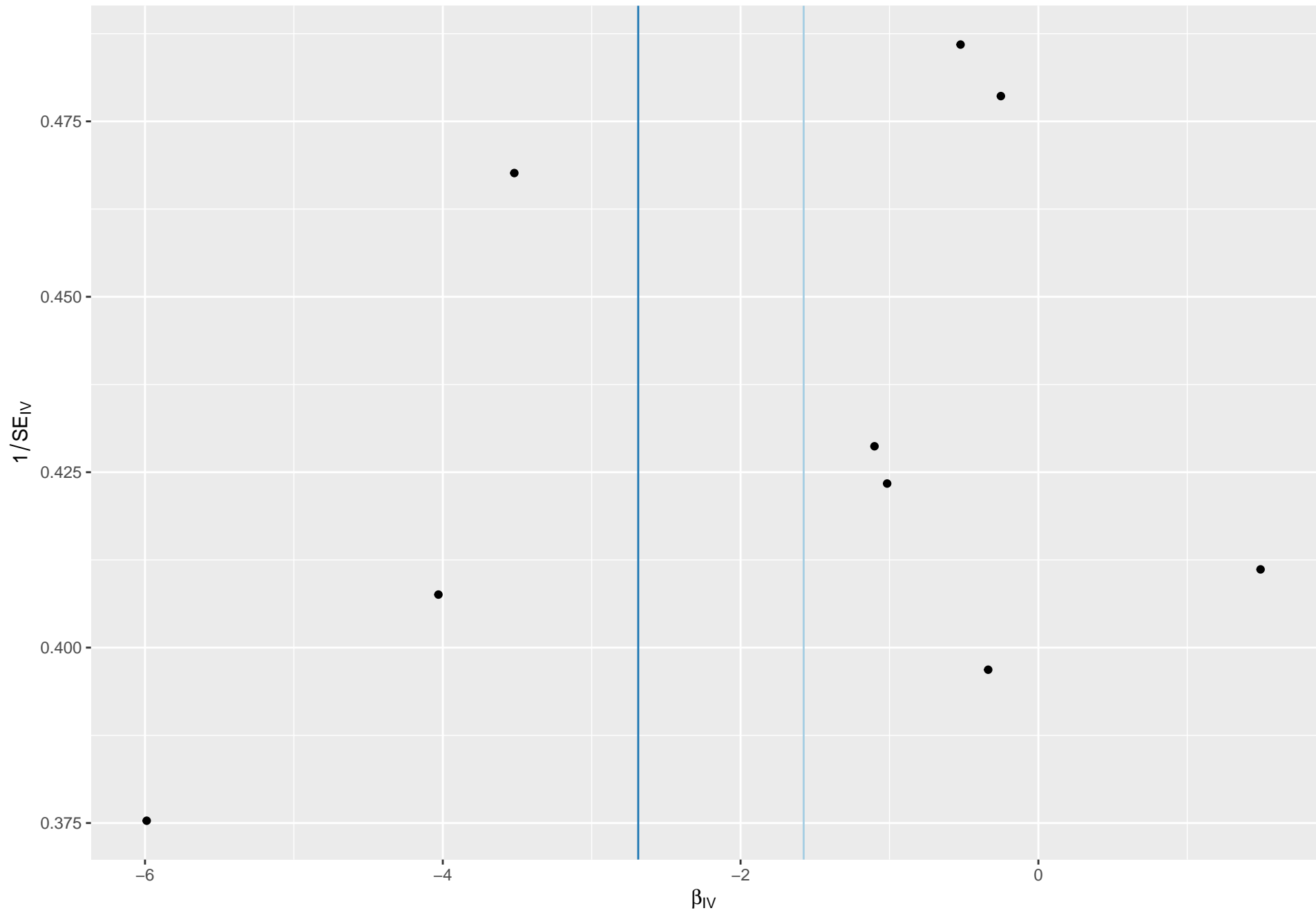

Supplement: Supplementary file 1 [file Data_Sheet_1.ZIP › Supplemenary Materials/Supplemenary Materials 4/unknowngenus.pdf]
